# Lessons from Nature for Carbon‐Based Nanoarchitected Metamaterials

**DOI:** 10.1002/smsc.202200039

**Published:** 2022-11-13

**Authors:** Jun Cai, Haoyu Chen, Youjian Li, Abdolhamid Akbarzadeh

**Affiliations:** ^1^ Department of Bioresource Engineering McGill University Montreal QC H9X 3V9 Canada; ^2^ Department of Mechanical Engineering McGill University Montreal QC H3A 0C3 Canada

**Keywords:** bioinspiration, carbon-based materials, molecular dynamics, nanoarchitected metamaterials

## Abstract

Bioinspired materials often achieve superior mechanical properties owing to their microscale architectures that resemble design motifs in biological materials. The bioinspired architectures can be extended to nanoscale, where carbon‐based materials, including graphene and carbon nanotubes, are excellent candidates as building blocks. This study introduces carbon‐based nanoarchitected metamaterials inspired by seven biological design motifs, i.e., cellular, gradient, tubular, fibrous, helicoidal, suture, and layered structures. Numerical studies based on molecular dynamics simulation along with continuum‐based finite element analysis are conducted for each bioinspired design to examine the unique mechanical properties, namely specific stiffness, specific strength, failure strain, and specific energy absorption, under tensile/shear loading conditions. Different deformation and failure mechanisms found by molecular simulation and continuum mechanics are discussed. The numerical results show that the mechanical properties of the introduced bioinspired and carbon‐based nanoscale designs may surpass the performance of the conventional carbon‐based counterparts. The developed nanoarchitected metamaterials demonstrate instances of possibilities for filling the empty regions in the Ashby charts to attain lightweight advanced materials that can also break the trade‐off between strength and failure strain. These findings impart lessons from the constitutive structure of biological materials to form the next generation of multifunctional architected metamaterials with rationally designed nano‐architectures.

## Graphical abstract

Carbon‐based nanoarchitected metamaterials inspired by seven biological design motifs are introduced. A comprehensive numerical study is carried out, and the mechanical merits of each design are examined. The developed metamaterials demonstrate possibilities for filling the empty regions in the Ashby charts. These findings shed light on design strategies for the next generation of multifunctional architected materials.

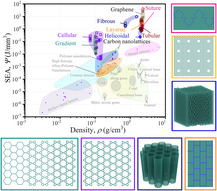

## 1 Introduction

Graphene, the classic one‐atom‐thick two‐dimensional (2D) material with hexagonal lattice of sp^2^‐bonded carbon atoms (a carbon atom bound to three atoms is sp^2^ hybridized. When carbon is bonded to four other atoms, the hybridization is sp^3^), was first theoretically discovered by Wallace^[^ ^1^ ^]^ in 1947 and then was experimentally realized around 1970s.^[^ 2, 3 ^]^ Another form of carbon atoms, known as one‐dimensional (1D) carbon nanotube (CNT), was uncovered later in 1991^[^ ^4^ ^]^ and reported to be made out of a single graphene layer later in 1993.^[^ 5, 6 ^]^ Since being discovered, these carbon‐based materials have attracted intensive research interest due to their outstanding thermo‐electro‐mechanical properties.^[^ 7, 8, 9, 10, 11 ^]^ For instance, the monolayer graphene exhibits a unique combination of ultrahigh mechanical properties (Young's modulus of ≈1 TPa and tensile strength of ≈130 GPa^[^ ^10^ ^]^), high thermal conductivity (3000–5000 W m^−1^ K^−1^ for suspended monolayer graphene),^[^ 8, 12 ^]^ and high electrical conductivity (10^4^ to 10^5^ S m^−1^).^[^ 13, 14 ^]^ The unique properties of graphene and CNT make them exciting candidates in flexible electronic devices,^[^ 15, 16 ^]^ nanosensors,^[^ ^17^ ^]^ complementary metal‐oxide‐semiconductor,^[^ ^11^ ^]^ and nanoelectromechanical systems (NEMS).^[^ ^18^ ^]^ Nevertheless, some of CNT's and graphene's intrinsic properties, such as the limited stretchability (≈0.2)^[^ 19, 20 ^]^ and low dimensional (1D and 2D) features,^[^ ^21^ ^]^ can hamper their applications.

By introducing rationally designed underlying architectures as the constitutive unit cell, architected materials, so‐called metamaterials, with unparalleled multiphysical properties can be developed at multiple scales, from nano to macro.^[^ 22, 23, 24, 25, 26, 27, 28, 29 ^]^ For example, 3D nanoarchitected pyrolytic carbon exhibits extreme energy dissipation, which is ≈70% superior to that of Kevlar composites and nanoscale polystyrene films, owing to its material architecture and nanoscale material size effects.^[^ ^23^ ^]^ Recent advances in additive manufacturing (AM) have emerged as a frontrunner for the fabrication of architected metamaterials to construct arbitrary complex architectures in a wide range of length scales. For instance, the feature resolutions of the architectures can be below 100 nm by utilizing photolithography.^[^ ^30^ ^]^ Hence, to fully leverage these new manufacturing techniques, it is essential to design and computationally optimize materials architectures at different length scales, especially nano‐ and atomic scales, to achieve desired properties. Two main strategies to date have been utilized to construct nanoarchitected metamaterials based on graphene sheets and CNTs by computational simulations. First, by employing various junctions to connect CNTs or curved graphene sheets covalently,^[^ ^31^ ^]^ lattice nano‐architectures (e.g., simple cubic (SC) and face‐centered cubic (FCC) lattice‐like hollow nanotruss^[^ 21, 32 ^]^) and graphene foams (e.g., graphene foams with triply periodic minimal surfaces^[^ ^33^ ^]^) can be developed. Another design strategy is inspired by the old Chinese and Japanese art of paper cutting and folding,^[^ 34, 35 ^]^ known as kirigami and origami (“kiri” means cut, “ori” means fold, and “kami” stands for paper),^[^ ^36^ ^]^ which can exploit local elastic instability to induce complex 3D patterns or structural reconfiguration upon mechanical deformations to realize shape‐changing micro/nanoscale metamaterials.^[^ 34, 37, 38, 39, 40, 41 ^]^ However, these graphene‐ and CNT‐based metamaterials still suffer from some weaknesses. For example, the stretchability of lattice‐like CNT nanotrusses is limited, and their strength shows a remarkable reduction compared to a pristine CNT.^[^ ^21^ ^]^ Graphene kirigami sheets exhibit excellent stretchability, while their tensile strength is only around 10% of the pristine graphene.^[^ ^20^ ^]^ Hence, in efforts to fully exploit the unrivalled properties of polymorphs of carbon, new topological design strategies should be developed for the next generation of multifunctional nanoarchitected metamaterials.

**Figure 1:**
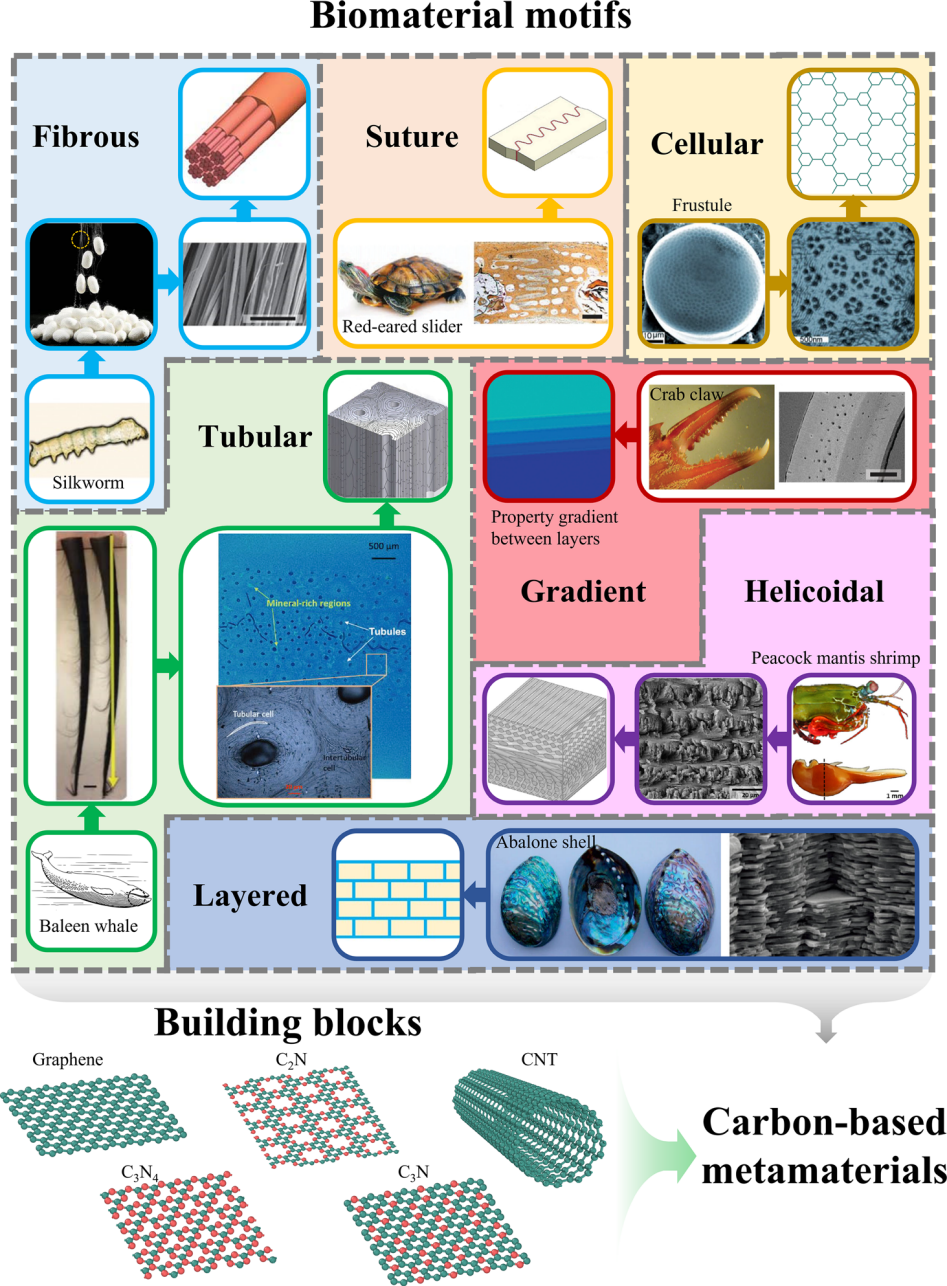
Seven biomaterial motifs used here for bioinspiration. Carbon‐based materials (e.g., graphene and CNT) can be utilized as building blocks to construct bioinspired metamaterials with alternative nanoarchitected motifs. Schematic graphs of fibrous, suture, and gradient design: Reproduced with permission.^[^ ^46^ ^]^ Copyright 2015, Wiley‐VCH. Graphs of crab claw: Reproduced with permission.^[^ ^92^ ^]^ Copyright 2009, Elsevier. Graphs of whale baleen: Reproduced with permission.^[^ ^82^ ^]^ Copyright 2018, Wiley‐VCH. Graph of silkworm: Reproduced with permission.^[^ ^93^ ^]^ Copyright 2008, Elsevier. Silk microscopy image: Reproduced with permission.^[^ ^94^ ^]^ Copyright 2008, Elsevier. Graphs of slider: Reproduced with permission.^[^ ^95^ ^]^ Copyright 2012, Elsevier. Graphs of slider shell: Reproduced with permission.^[^ ^96^ ^]^ Copyright 2009, Elsevier. Graphs of frustule: Reproduced with permission.^[^ ^83^ ^]^ Copyright 2009, Wiley‐VCH. Graphs of helicoidal design: Reproduced with permission.^[^ ^84^ ^]^ Copyright 2014, Elsevier. Graphs of abalone shell are public domain images from Wikimedia Commons, attributed to Doka54 (left) and Fabian Heinemann (right).

Biological materials, having undergone protracted natural selection, feature remarkable mechanical properties (e.g., high specific strength and toughness), and specific multifunctionalities, (e.g., self‐healing,^[^ ^42^ ^]^ photonic bandgap,^[^ ^43^ ^]^ and structural multistability^[^ ^44^ ^]^) owing to their delicately arranged nano/microstructures. Research on biological materials provides a critical repository of inspiration, and thereby generates architectural design rationale for the engineering of advanced materials.^[^ ^45^ ^]^ Despite around seven million animal species that are living on the earth, there is notable repetition in the structures of diverse biological materials.^[^ 45, 46 ^]^ Eight structural design elements have been identified as the most common elements among a variety of biological materials: cellular, gradient, helical, fibrous, layered, tubular, overlapping, and suture.^[^ ^46^ ^]^ Their structural characteristics, along with mechanical advantages, are listed in **Table** **1**. We should note that the overlapping motif contains individual members that slide on each other, which may not be employed in nanoarchitected metamaterials with current modeling approaches. Moreover, the utilized design motifs do not encompass either the shape morphing mechanisms found in nature (e.g., in Venus flytrap, Impatiens glandulifera seedpods, and Dionaea muscipula leaves^[^ 47, 48 ^]^) that have inspired engineers to realize shape‐changing metamaterials. The microscopy images along with the conceptual bioinspired models for seven design motifs used in this perspective study are shown in **Figure** **1**. By inspiring from the nano/microstructure of these design motifs, architected materials can achieve significantly improved mechanical properties (e.g., excellent strength and toughness). It is worth mentioning that toughness and energy absorption terms have been interchangeably used in the bioinspiration literature.^[^ 42, 43 ^]^ For example, helicoidal‐inspired carbon fiber‐reinforced composites (CFRC) exhibit much better impact and crack resistance than traditionally laminated CFRC.^[^ 49, 50 ^]^ In spite of recent investigation, the bioinspired architectures at the nanoscale have not been systematically studied, remaining questions if and how the bioinspired architectures constructed out of 1D or 2D nanoscale materials can realize enhanced material property improvement and even offer counterintuitive multifunctional performance enhancement.

**Table 1:** Design motifs commonly found in biological microstructures along with their characteristics and enhanced mechanical properties of the associated bioinspired materials

Design motifs	Characteristics	Enhanced mechanical properties
Cellular	High porosity, foam architecture	Specific strength, energy absorption^[^ 46, 86 ^]^
Gradient	Nonuniform material distribution and interfaces	Toughness^[^ ^87^ ^]^
Tubular	Organized pores oriented in the out‐of‐plane direction	Energy absorption^[^ ^82^ ^]^
Fibrous	Highly aligned fiber‐like structures	Tensile strength^[^ ^46^ ^]^
Helicoidal	Sequential rotation of highly anisotropic layers along the out‐of‐plane direction	Toughness^[^ 88, 89 ^]^
Layered	Composite of soft and hard phases	Strength and toughness^[^ 90, 91 ^]^
Suture	Stiff components connected by interlocking seams	Strength and energy absorption^[^ ^46^ ^]^
Overlapping	Flexible protective surfaces composed of individual plates or scales	Penetration resistance and flexibility^[^ ^46^ ^]^

**Figure 2:**
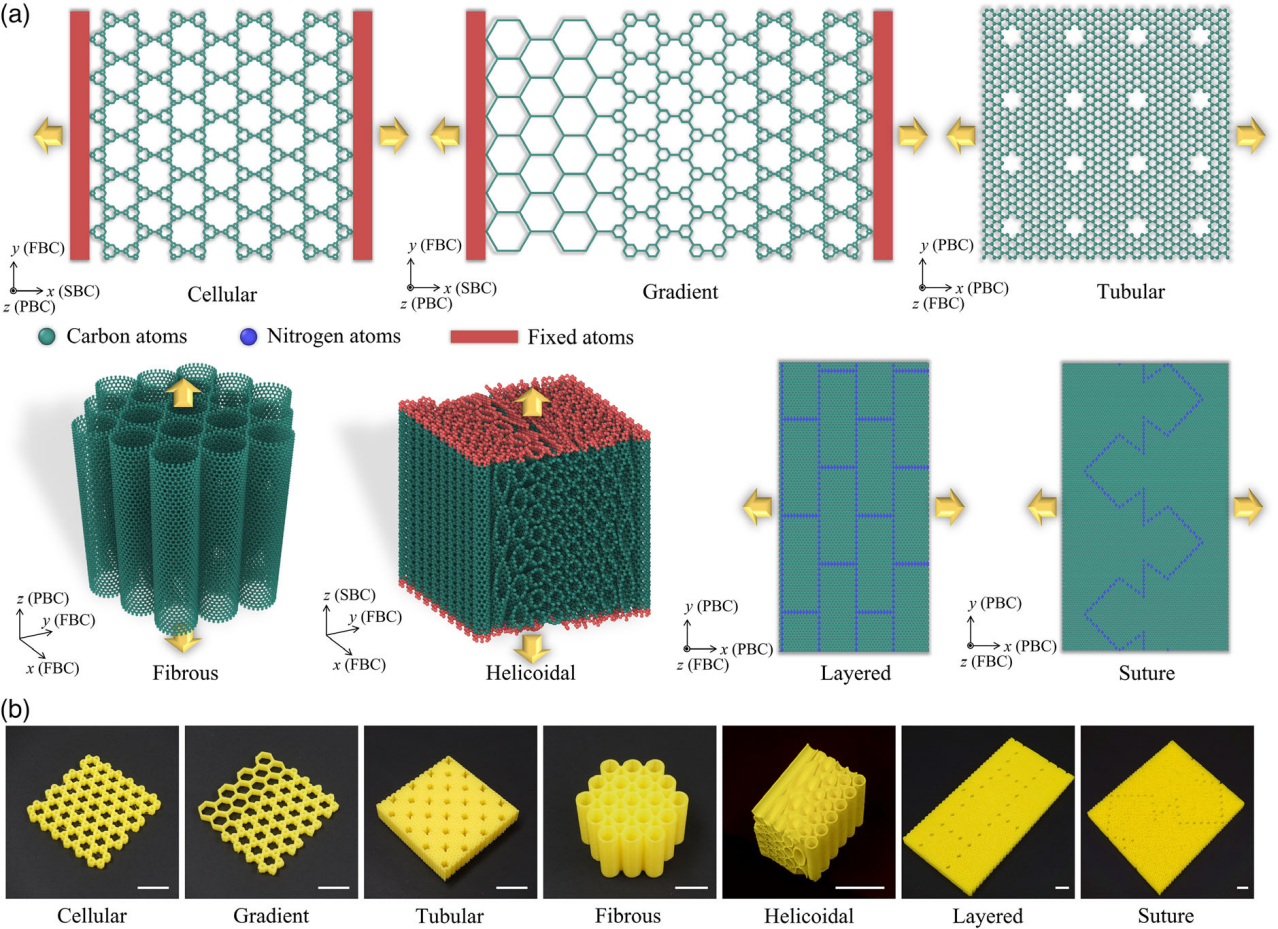
Bioinspired designs. a) Bioinspired carbon‐based metamaterials with the imposed boundary conditions used in MD simulation. PBC, FBC, and SBC represent periodic, fixed, and shrink‐wrap boundary conditions, respectively. b) Seven bioinspired designs 3D printed out of poly(lactic acid) (PLA) (scale bar: 10 mm).

Herein, we focus on bioinspired architected metamaterials composed of low‐dimensional nanomaterials (i.e., graphene and CNT). We propose seven groups of bioinspired architectural designs by resorting to the biological motifs and carbon‐based material construction principles, as shown in **Figure** **2**a. For the visualization of the bioinspired designs, 3D printed prototypes are also fabricated and displayed in Figure 2b. Through computational simulations using molecular dynamics (MD) and finite element method (FEM), we evaluate their mechanical properties and elicit their elastic‐to‐failure deformation mechanisms in molecular and continuum mechanics. Finally, we conduct a holistic comparison among the all introduced nanoarchitected designs in terms of specific stiffness, strength, toughness, and stretchability. The mechanical merits of the bioinspired carbon‐based nanoarchitected metamaterials are compared with the commonly used natural and engineering materials. The present paper is organized as follows: Section 2 presents the details of computational simulation approaches. In Section 3, seven bioinspired graphene/CNT‐based metamaterials are introduced, followed by the investigation of their mechanical properties and deformation mechanisms. To better evaluate their mechanical performance, material property charts are provided in Section 4. A detailed discussion about the difference between MD and FEM simulations, other 2D nanomaterials candidates, and multiphysical properties of bioinspired designs is also presented. Finally, Section 5 highlights the main conclusions extracted from this study.

## 2 Modeling and Methods

### 2.1 Molecular Dynamics Simulation

All atomic simulations are conducted using the large‐scale atomic molecular massively parallel simulator (LAMMPS).^[^ ^51^ ^]^ Open Visualization Tool (OVITO)^[^ ^52^ ^]^ is employed to visualize the evolution of atomic structures. AIREBO^[^ ^53^ ^]^ potential function is used to describe C–C interatomic interactions. The cutoff distance between C–C is set as 2.0 Å to avoid the spurious strengthening effect.^[^ ^54^ ^]^ For layered‐ and suture‐inspired graphene metamaterials with nitrogen atoms, Tersoff potential^[^ ^55^ ^]^ is applied to model the cross‐interactions between carbon and nitrogen atoms. The integration time step is set as 1.0 femtoseconds (fs). The boundary conditions are shown in Figure 2. For cellular‐ and gradient‐inspired designs, the periodic boundary condition (PBC) is applied to the out‐of‐plane (*z*‐direction) direction; fixed boundary conduction (FBC) is used in the in‐plane transverse direction (a virtual box enveloping all the boundaries is defined. The dimension of the box in the in‐plane transverse direction should be large enough to allow deformation); shrink‐wrap boundary condition (SBC; the shrink‐wrap boundary means that the position of the face is set to encompass the atoms in that direction, which is a non‐PBC in MD simulation) is applied in the loading direction. For fibrous‐inspired CNT bundles, the *z*‐direction is periodic, while the *x*‐ and *y*‐directions are fixed. PBCs are applied to the planar directions (*x*‐ and *y*‐directions), and an FBC is used in out‐of‐plane direction (*z*‐direction) in tubular‐, layered‐ and suture‐inspired designs. For helicoidal designs, FBCs are applied in the *x*‐ and *y*‐directions; SBC is applied in the z‐direction. Energy minimization is first performed by the conjugate gradient algorithm to obtain stable graphene‐ and CNT‐based nanostructures. Then, all systems are relaxed in the isothermal‐isobaric ensemble (NPT) for 30 picoseconds (ps). For cellular‐, gradient‐, and helicoidal‐inspired models, the bottom layers of the carbon atoms are frozen, and the top layers of carbon atoms are given a constant velocity to simulate the uniaxial tensile deformation. In the other bioinspired designs, the samples are subjected to a homogenous tensile deformation by rescaling the vertical coordinates of all atoms in the virtual simulation box. The strain rate is set at $1\times 10^{9}$ s^−1^ in all samples. The axial stress is calculated by averaging the virial stress of all free atoms in the system as

(1) $$\sigma_{ij}=\frac{1}{V_{0}}\sum_{\gamma =1}^{\gamma =n}S_{ij}^{\gamma }$$

where $V_{0}$ is the initial volume of the system (the calculation of $V_{0}$ can be found in Supporting Information S1, Supporting Information); $S_{ij}^{\gamma }$ is the stress tensor for atom *γ*; and *i* and *j* denote indices in the Cartesian coordinate system (*i*, *j* = 1, 2, 3). The atomic stress of individual carbon atom *α* in the graphene‐ and CNT‐based nanostructures is calculated by the following formula

(2) $$S_{ij}^{\alpha }=\frac{1}{2}m^{\alpha }v_{i}^{\alpha }v_{j}^{\alpha }+\sum_{\beta =1}^{\beta =n}r_{\alpha \beta }^{\;j}f_{\alpha \beta }^{i}$$

where $m^{\alpha }$ and $v^{\alpha }$ denote the mass and velocity of atom *α*; $r_{\alpha \beta }$ and $f_{\alpha \beta }$ are the distance and force between atoms *α* and *β*.

### 2.2 Finite Element Analysis

Equivalent FEM models for all cellular‐ and gradient‐inspired designs are built. FEM simulation is performed to investigate the quasi‐static tensile behaviors of these models under the framework of solid mechanics. The geometries of the designed atom arrangements are converted into equivalent solids to form the geometries of FEM models. For cellular‐inspired designs, a converted solid cell wall has the same length and width as those of an envelope cuboid of the graphene‐based cell wall, while the effective thickness is defined as described in Section 2.3. These equivalent FEM models can accurately inherit the elastic behaviors of the corresponding MD models. The modeling procedure for tubular‐ and helicoidal‐inspired designs can be found in Supporting Information S2 and S3, Supporting Information.

A commercial FEM code (LS‐DYNA) is utilized. In the FEM models, beam elements (BEAM 161) are used to simulate the numerous members in bioinspired architected materials. After a convergence analysis of accuracy against the number of elements, each 1D member (i.e., cell wall or strut) is modeled by at least four beam elements. Hughes‐Liu beam element integration formulation is applied with 2 × 2 integration points. Elastic‐failure material properties, including Young's modulus, Poisson's ratio, and failure strain, are defined. These material properties are inherited from MD simulations through an equivalent method illustrated in Section 2.3. For cellular‐inspired models, translational ($\overset{↔}{z}$) and rotational ($\hat{xz}$ and $\hat{yz}$) degrees of freedom (DoFs) are constrained for all the beam elements to ensure the plane strain condition in the *x*–*y* plane. All DoFs are constrained for the nodes at one edge of the model, while only the translational DoF along the loading direction is released at the opposite edge, where a prescribed motion with uniform speed is applied. A strain rate of 0.05 s^−1^ is adopted for a quasi‐static loading. To validate the accuracy of the abovementioned FEM model, numerical computation is carried out on three 1st order honeycomb materials with different cell wall lengths in Supporting Information S4, Supporting Information. The stiffness from FEM simulation agrees well with theoretical predictions and MD results. For tubular‐ and helicoidal‐inspired designs, we have attempted to build equivalent FEM models with moderate accuracy to catch the elastic behaviors from the MD results, as presented in Supporting Information S2 and S3, Supporting Information. In the future, equivalent models with a modified beam theory can more accurately describe the mechanical behaviors of C—C bonds.

The mechanical properties, including specific stiffness, specific strength, failure strain, and specific energy absorption (SEA) of all designs, are evaluated by MD and FEM simulations. The specific stiffness, specific strength, and SEA are expressed by

(3a) $$\text{Specific strength}:\;\bar{E}=\frac{E}{\bar{\rho }}$$

(3b) $$\text{Specific strength}:\;\bar{\sigma }=\frac{\sigma_{u}}{\bar{\rho }}$$

(3c) $$\text{SEA}:\;\Psi =\frac{\int \sigma d\varepsilon }{\bar{\rho }}$$

(3d) $$\text{Gravimetric specific strength}:\;\bar{\sigma }=\frac{\sigma_{u}}{\rho }$$

where *E*, $\sigma_{u}$, *σ*, *ρ*, and $\bar{\rho }$ denote the Young's modulus, ultimate tensile stress, tensile stress, density, and relative density, respectively.

### 2.3 Effective Parameters Applied in FE Analysis

The material parameters obtained by MD simulation cannot be directly used in the FE analysis because the graphene nanosheets and CNT contains the thickness effect.^[^ 20, 56, 57 ^]^ The 2D graphene nanosheets are one‐atom‐thick nanomaterials. In MD calculation, we assume a thickness of 0.34 nm for all samples.^[^ 20, 39, 41 ^]^ To exclude the thickness effects from these material parameters (e.g., stiffness and ultimate strength), an effective thickness for graphene and CNT is identified based on the strain energy and deflection in a three‐point bending simulation.^[^ 56, 57 ^]^ The effective thickness and stiffness used in FE calculation are 1.27 Å and 2.81 TPa, respectively.^[^ 20, 57 ^]^

Based on the equivalent thickness of 3.4 Å used in the MD simulation, the equivalent volume of individual carbon atoms in the pristine graphene and CNT is calculated as 8.92 Å^3^. Then the relative density of the designed graphene‐ and CNT‐based nanostructures is calculated by $\bar{\rho }=n/\left(V_{0}/8.92\right)$, in which *n* and $V_{0}$ are the number of atoms and the volume in the designed nanostructures, respectively. The relative density of all samples studied in this work is shown in Table S1, Supporting Information.

## 3 Results

### 3.1 Cellular‐Inspired Design

**Figure 3:**
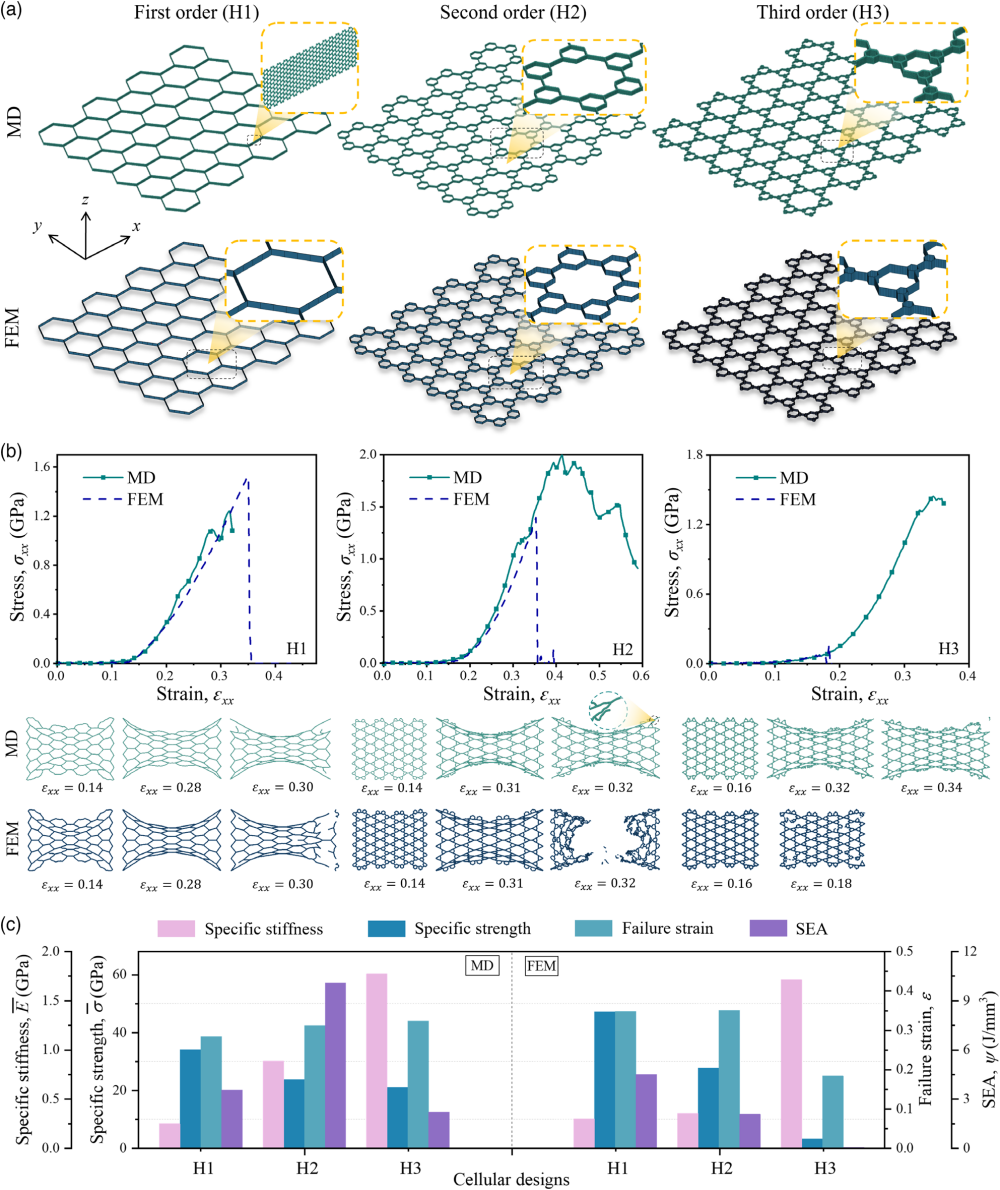
Cellular‐inspired graphene metamaterial. a) Schematic of hierarchical graphene metamaterials including regular honeycomb (1st order), 2nd order, and 3rd order (from left to right) constructed in MD and FE simulations. b) Stress–strain curves of cellular‐inspired graphene metamaterials under a tensile load in the *x*‐direction obtained by MD and FE simulations, and the corresponding configurations at certain tensile strains. c) Mechanical properties (specific stiffness, specific strength, SEA, and failure strain) of cellular‐inspired graphene metamaterials obtained by MD and FE simulations.

Cellular biomaterials commonly possess hierarchical structures, which bring cellular designs into the constructing members at successive levels from the structural level to the molecular one. In this section, inspired by the hierarchical concept, two different designs, i.e., 2nd order and 3rd order honeycomb (H2 and H3), are designed. In comparison, regular (H1) honeycomb is selected to represent conventional cellular materials. Their geometric parameters are shown in Supporting Information S6. Both MD and FEM numerical models are constructed via the abovementioned methods, as presented in **Figure** **3**a. It is worth mentioning that all carbon atoms in the honeycomb nanostructures are connected covalently. The stress–strain response of the base material (graphene) for constructing cellular‐inspired designs is shown in Supporting Information S7, Supporting Information. Another cellular‐related pomelo peel‐inspired design is shown in Supporting Information S8, Supporting Information.

From Figure 3b, the stress–strain curves of MD simulations can be generally divided into four periods, i.e., linear region (LR), large deformation region (LDR), post‐collapse increase region (PCIR), and post‐collapse decrease region (PCDR). For all three designs, first, the stress increases with a low and approximately constant slope along with some fluctuations during the LR. Then, the stress–strain curves rise sharply in the LDR followed by a minor decrease due to the collapses of a small amount of sp^2^ bonds. The exponential increase of stress is due to the gradual straightening process of the architectural members at two side edges. The collapses arise near the two side edges and the loading edge. After that, there is a post‐collapse increase period of stress on the curve accompanied by fluctuations due to the continued straightening process until the major propagation of the fractures. Finally, in the last region, the stress–strain curves fall gradually, as the fracture propagation sustains until the cellular metamaterials completely lose the bearing capability. Similarly, the stress–strain curves of FEM simulations have three periods, namely LR, LDR, and post‐collapse region (PCR). These curves are nearly consistent with their MD counterparts during the LR and the early stage of the LDR. However, the endpoints of the LDR are different from those of MD results, which is due to the difference in the failure modes between the two numerical approaches. For the H1 and H2, the FEM model exhibits better stretchability than the atomic model. H3 shows premature collapses just after the LDR begins (*ε*_*xx*_ = 0.184), resulting in much lower strength and energy absorption properties than the atomic model. In the FEM results, a rapid fracture happens after a large deformation as the raw material possesses elastic‐brittle properties. In comparison, a progressive collapse happens in the MD models. Lastly, in the PCR of FEM, the stress rapidly decreases to zero after the brittle collapse.

The mechanical properties, including specific stiffness, specific strength, failure strain, and SEA of all the designs, are summarized and compared in Figure 3c. For the specific stiffness, from both FEM and MD results, bioinspired metamaterials show an increasing trend as the hierarchical level increases. The hierarchical designs realize a ≈6.5 times improvement of specific stiffness compared with the nonhierarchical one. The difference in value between the two simulation methods can be explained by slope measuring error when the curves fluctuate. This problem can possibly be solved by decreasing the loading rate in MD and FEM simulations. For the specific strength, the trend remains the same for both numerical methods, while the decline with the increased hierarchy is more significant in FEM results (for MD results, the specific strength of H3 and H2 is 61.7 and 69.6 percent of H1, respectively, while the corresponding ratios are 6.98 and 58.8 percent for FEM results). In terms of the failure strain, the H2 and H3 prevail over the conventional design at the atomic level, while only the H2 surpasses the baseline by a minimal advantage from FEM results. For the SEA, the H2 surpasses the H1 by 180%, while the H3 one degenerates to 65% of the conventional design. Both hierarchical designs (H2 and H3) present worse SEAs than the conventional (H1) one in the continuum mechanics framework. Moreover, we simulate the cellular‐inspired graphene metamaterials with PBCs applied in three orthogonal directions; the numerical results are presented in Supporting Information S9, Supporting Information.

### 3.2 Gradient‐Inspired Design

**Figure 4:**
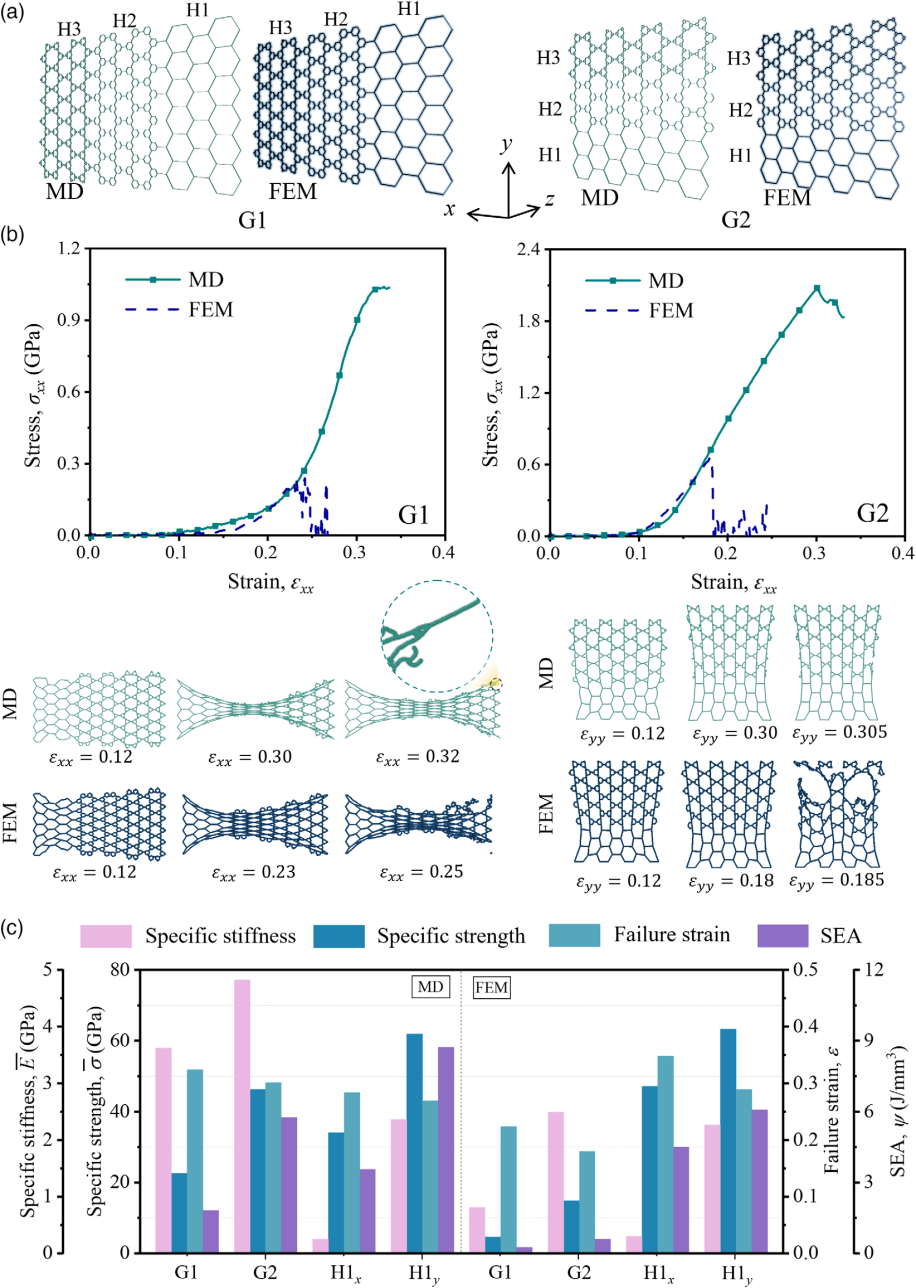
Gradient‐inspired graphene metamaterial. a) Schematic of graphene cellular metamaterials arranged in a sequence from 1st order to 3rd order to form a gradient relative density distribution from 0.0321 to 0.0632 in MD and FE simulations. Gradient design 1 (G1) has a gradient orientation along the *x*‐direction, while gradient design 2 (G2) is arranged along the *y*‐direction. b) Stress–strain curves of gradient‐inspired graphene metamaterials under a tensile load along the gradient orientations obtained by MD and FE simulations and the corresponding configurations at certain tensile strains. c) Mechanical properties (specific stiffness, specific strength, SEA, and failure strain) of gradient‐inspired graphene metamaterials obtained by MD and FE simulations.

Hierarchical designs in Section 3.1 are arranged in a sequence from 1st order to 3rd order to form a gradient relative density distribution from 0.0321 to 0.0632. As a result, gradient design 1 (G1) has a gradient orientation along the *x*‐axis, while gradient design 2 (G2) is arranged along the *y*‐axis. MD and FEM models are established as presented in **Figure** **4**a. Both models are under tensile loading along the gradient orientation. The stress–strain curves and deformation histories are illustrated in Figure 4b. The MD and FEM curves can also be divided into three and four periods, respectively, namely the same as the period division for cellular metamaterials. From the deformation plots in Figure 4b, the stretching deformation is generally nonuniform. The large deformation first takes place in the low relative density area and then propagates to the other end. Figure 4c compares the mechanical properties of gradient designs with those of the conventional cellular design under corresponding loading directions. Specifically, G1 and G2 are compared with H1 loaded in the *x*‐ and *y*‐directions, respectively. The difference of mechanical properties between the two numerical approaches is also described in this graph. For atomic models, both the specific stiffness and failure strain are greater than the corresponding conventional counterparts. The gradient design G1 can realize up to 15 times improvement of specific stiffness and 14% higher failure strain. However, the specific strength and SEA are not as high as those of the baselines due to the introduction of the 3rd order cellular design, which possesses poor performance of these properties. For FEM models, the improvement in terms of specific stiffness is not as significant as the MD results. Regarding the specific strength, failure strain, and SEA, the gradient designs in the continuum mechanics framework show no advantage compared with H1. In Figure 4, the gradient‐inspired graphene metamaterials are constructed with three layers of each building block (H1, H2, and H3). To exclude the edge effect induced by the limited layers of H1, H2, and H3, gradient‐inspired graphene metamaterials with five and seven layers of H1, H2, and H3 are constructed in Supporting Information S10, Supporting Information. The stress–strain responses and deformation processes indicate that the number of layers shows little effect on the mechanical properties and deformation of gradient‐inspired graphene metamaterials.

### 3.3 Tubular‐Inspired Design

**Figure 5:**
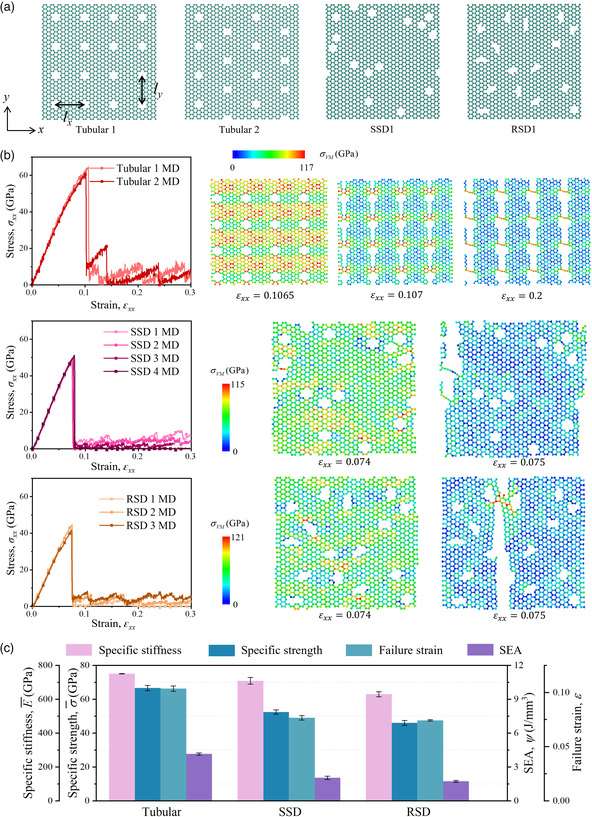
Tubular‐inspired graphene metamaterial. a) Schematic of tubular‐inspired graphene metamaterials (Tubular 1 and Tubular 2) and the graphene sheets with standard shape distribution (SSD) and with random shape distribution (RSD). b) Stress–strain curves of tubular‐inspired graphene metamaterials and samples in SSD and RSD group under a tensile load in the *x*‐direction obtained by MD and the corresponding configurations at certain tensile strains. The contour indicates the von Mises (VM) stress. c) Mechanical properties (specific stiffness, specific strength, SEA, and failure strain) of tubular‐inspired graphene metamaterials, SSD and RSD obtained by MD simulation.

Inspired by the tubular structures, we modify graphene by introducing tubes to tune its mechanical properties. **Figure** **5**a shows the atomic configurations of graphene metamaterials for MD simulation. Tubular 1 and Tubular 2 represent two designs inspired by tubular structures, where the regular tubules are distributed uniformly in pristine graphene. For comparison, graphene sheets with randomly distributed tubules are constructed: tubules in standard shape distribution (SSD) and tubules in random shape distribution (RSD). The detailed designs of the SSD and RSD groups are illustrated in Supporting Information S11, Supporting Information. In addition, the tubules are formed by deleting six carbon atoms nearby; hence, the relative densities are $\bar{\rho }$ = 0.9464 for all samples. In Figure 5b, we report the stress–strain curves of tubular inspired, SSD, and RSD samples under a tensile load in the *x*‐direction. The mechanical responses of all three groups with *y*‐direction tension can be found in Supporting Information S12. The SSD and RSD samples show a typical brittle fracture, while a zigzag‐like stress–strain response is observed after the yielding in tubular‐inspired graphene metamaterials. Further understanding of the mechanisms resulting in the different responses of tubular, SSD, and RSD samples can be obtained by inspecting the numerical snapshots of the corresponding designs (Figure 5b). The color of the configurations in MD represents the von Mises stress distributions. It can be noted that stress is concentrated around the tubules in all MD samples, resulting in the initial nucleation of crack followed by a sharp stress decline. Due to the ordered distribution of tubules in the tubular design group, the stress distribution is more uniform compared to the samples in SSD and RSD groups. After the initial crack nucleation, the crack propagates through the samples quickly in SSD and RSD groups, leading to a brittle fracture. However, in tubular‐inspired designs, such cracks propagate across the ordered distributed tubules, leaving a slight connection between the fractured graphene sheets (*ε*_*xx*_ = 0.2 in Tubular 1). The same deformation process can be found in Tubular 2 in Supporting Information S12, Supporting Information. Such slight connections work like fibers with weak bonds between carbon atoms, which are broken one by one with increased tensile strains, resulting in the zigzag‐like stress response after yielding. Next, to better compare the mechanical properties of the tubular‐inspired, SSD, and RSD designs, the specific stiffness, specific strength, SEA, and failure strains of the corresponding samples in Figure 5b are presented in Figure 5c. Note that the samples in the same group do show similar stress–strain responses; the mechanical properties of each design shown in Figure 5c are the average values of all samples in the same group. The results reported in Figure 5c indicate that the tubular‐inspired graphene metamaterial is stiffer and stronger than SSD and RSD designs; simultaneously, the stretchability is also increased. We also conduct FEM simulations on the tubular‐inspired, SSD, and RSD samples in Supporting Information S2, Supporting Information. The numerical results show similar stress–strain responses and deformation mechanisms as observed in MD simulation.

### 3.4 Fibrous‐Inspired Design

**Figure 6:**
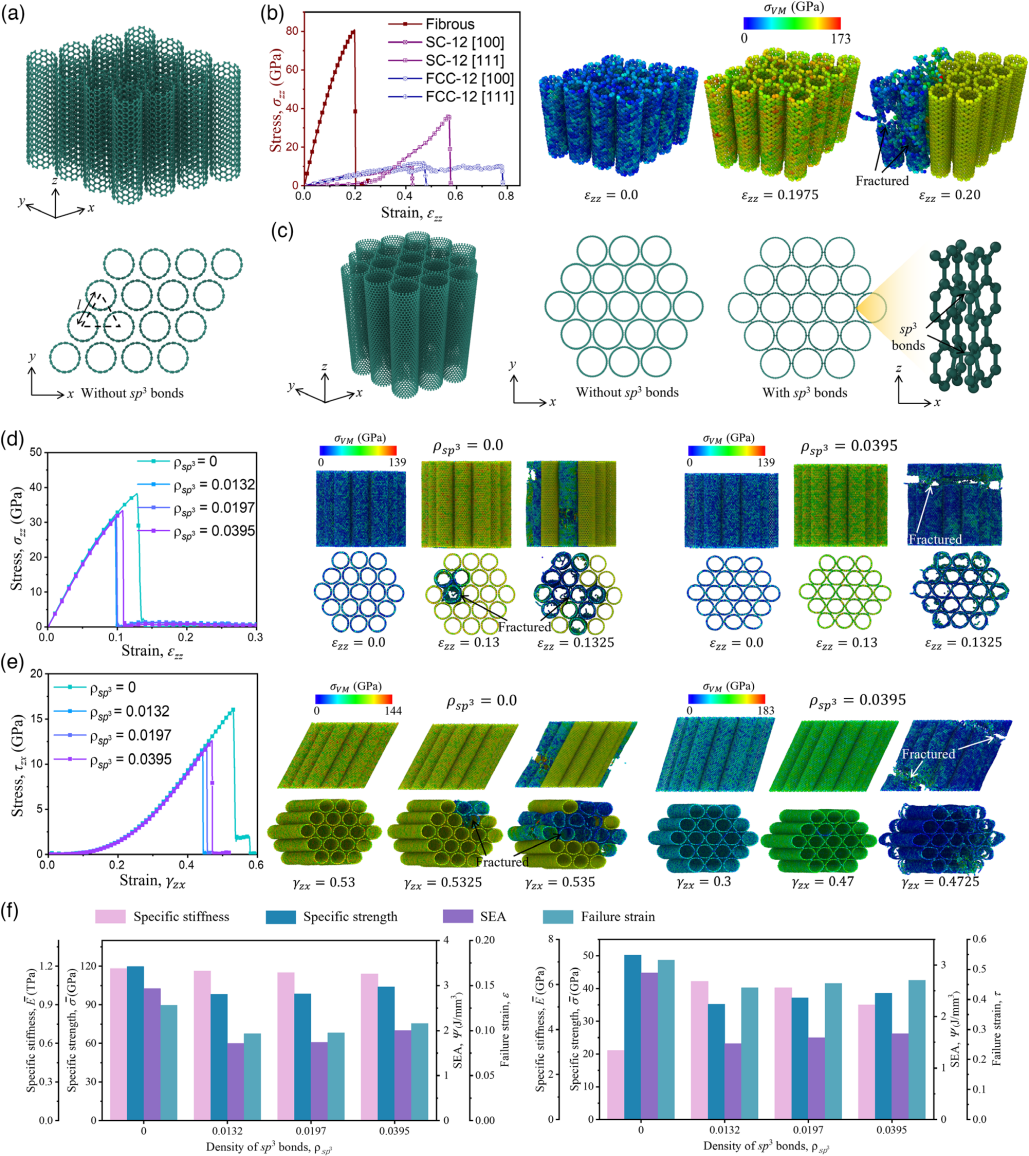
Fibrous‐inspired CNT metamaterial. a) Schematic of fibrous‐inspired CNT bundle without any bond connections between adjacent CNT filaments. b) The comparison between stress–strain responses of fibrous‐inspired CNT bundle and CNT simple‐cubic (SC) and face‐centered‐cubic (FCC) nanotruss (the indictors shown in the stress–strain curves can be find in Ref. [21]) subjected to uniaxial tension and the corresponding atomic configurations of fibrous‐inspired CNT bundle at certain strains. The contour displays the von Mises (VM) stress. c) Schematic of fibrous‐inspired CNT bundles (19 CNT filaments) with/without sp^3^ bonds between adjacent CNTs. d,e) The stress–strain curves of fibrous‐inspired CNT bundles characterized by different sp^3^ bond densities subjected to tension (d) and shearing (e), and the corresponding atomic configurations of fibrous‐inspired CNT bundle at certain strains. The contour displays the von Mises (VM) stress. f) Mechanical properties (specific stiffness, specific strength, SEA, and failure strain) of fibrous‐inspired CNT bundles characterized by different sp^3^ bond densities subject to tension/shearing.

Individual CNT shows exceptional mechanical properties at the nanoscale, used as reinforcement in composite materials. However, the mechanical properties (strength and stiffness) of individual CNT are strongly dependent on its direction. Hence, the arrangement of CNTs as reinforcement in composited materials is quite important, which decides the transformation efficiency of CNT mechanical properties to the composite materials. Biological materials that require high tensile strength and stiffness are often organized as fibrous structures with numerous aligned fibers in one direction,^[^ ^46^ ^]^ which are found in spider silk, hagfish slime, silkworm silk, and rat tendon.^[^ ^46^ ^]^ Inspired by biological materials, the structural element of fibrous is proposed to be applied in 3D CNT architected metamaterials to increase their strength and stiffness. **Figure** **6**a shows the fibrous‐inspired CNT bundles with 16 CNT filaments. To compare our fibrous‐inspired design with widely studied nanoarchitected CNT metamaterials, such as SC and FCC nanotrusses,^[^ ^21^ ^]^ the (8,8) single‐walled CNTs are selected as building blocks. Here, a close‐packed configuration with a triangular lattice is constructed (Figure 6a) because it has been widely adopted CNT bundle with the lowest system energy.^[^ ^58^ ^]^ We also construct a CNT bundle with 16 CNT filaments in a square lattice, which is found to transfer to a triangular lattice (similar to the one shown in Figure 6a) after the process of energy minimization and full relaxation (Supporting Information S13, Supporting Information). Another geometrical parameter for the CNT bundle is the interthread separation distance (*l*) between two CNT filaments, which refers to the distance between the centers of the two adjacent CNT filaments. The interthread separation distance is *l* = 14.043 Å for CNT (8,8) in this work identified by the relaxation processes under isothermal‐isobaric ensemble in MD simulation.^[^ ^58^ ^]^ Figure 6b compares the stress–strain responses of our design with SC and FCC CNT nanotrusses^[^ ^21^ ^]^ under tensile loading in the *z*‐direction. It should be emphasized that all five samples share the same approximate relative density. We found that the fibrous‐inspired CNT bundle shows higher stiffness and ultimate strength than SC and FCC CNT nanotrusses. As the representative deformation processes of the fibrous‐inspired CNT bundle shown in Figure 6b, the stress is distributed uniformly and accumulates with the increased tensile strains; the earliest fracture occurs at the outer layer of the CNT bundle and transports quickly to the whole CNT bundle, acting as a brittle fracture. In nanoarchitected CNT metamaterials,^[^ 21, 32 ^]^ the stress concentration is found in the junctions of CNTs, and only partial CNTs participate in the force bearing, resulting in weak stiffness and strength. The failure strain of the fibrous‐inspired CNT bundle is 0.2 (Figure 6b), which is defined as the strain of the first fracture (also known as elastic limit). In Figure 6b, the FCC‐12 [100] sample is totally fractured at a tensile strain of 0.78; however, the initial fracture occurs at a strain of 0.2. Hence, the stretchability does not show any improvement compared with the fibrous‐inspired CNT bundles. The comparison of the mechanical properties indicates that the CNT bundle in fibrous topology could be an effective way to translate the exceptional properties of individual CNT to achieve high performance of 3D CNT architectures.

However, one of the main limitations of fibrous‐inspired CNT bundles, especially when manufacturing the microscopic fibrous CNT bundles, is the weak shear interactions between adjacent CNTs or within a multiwalled CNT.^[^ ^59^ ^]^ One promising approach to overcome this limitation is introducing the strong cross‐linking bonds between the CNTs.^[^ 59, 60, 61 ^]^ In Figure 6c, we construct fibrous‐inspired (28,0) CNT bundles with cross‐links characterized by different densities. Different cross‐links within CNT bundles have been reported, such as single benzene,^[^ ^62^ ^]^ double benzene,^[^ ^62^ ^]^ triple benzene,^[^ ^62^ ^]^ sp^3^ bonds,^[^ 63, 64 ^]^ and point defect (divacancy, Frenkel, and interstitial) bridging.^[^ 59, 60, 61 ^]^ The sp^3^ bonds are chosen here to study the effect of cross‐linking bonds on the mechanical behaviors of fibrous‐inspired CNT bundles. The initial CNT bundle is constructed with (28,0) CNTs arranged in a hexagon lattice where the distance between adjacent CNTs is 2.0 Å, which can prevent the sp^3^ bond formation but retain the van der Waals connection between adjacent CNTs. The sp^3^ bonds are added by incrementally moving two close atoms in adjacent CNTs from 2.0 Å to the sp^3^ bond distance (Figure 6c).^[^ ^63^ ^]^ Here, four different densities of sp^3^ bonds are considered ($\rho_{{\text{sp}}^{3}}$ = 0, 0.0132, 0.0197, and 0.0395), which are defined as the number of carbon atoms with sp^3^ bonds divided by the number of carbon atoms in the system. Figure 6d,e show the stress–strain responses of fibrous‐inspired CNT bundles characterized by different sp^3^ bond densities and the corresponding atomic deformations subjected to uniaxial tension and shearing, respectively. From the stress–strain responses, it is found that these samples exhibit the same deformation mechanism: the brittle broken after nonlinear elasticity. We then calculate the mechanical properties (specific stiffness, specific strength, SEA, and failure strain) of the fibrous‐inspired CNT bundles characterized by different sp^3^ bond densities under tension and shearing in Figure 6f. When the fibrous‐inspired CNT bundles are deformed under uniaxial tension, the specific stiffness, specific strength, SEA, and failure strain are decreased after introducing sp^3^ bonds between adjacent CNTs. It is because the CNT filaments in the CNT bundle without sp^3^ bonds are arranged in the same direction with the same length, indicating that all the CNT filaments can bear the tensile load. However, the CNT filaments cannot be of the same length when manufacturing the fibrous‐inspired CNT bundles; hence, the mechanical properties are dependent on the shear interactions between adjacent CNTs.^[^ ^59^ ^]^ Here, the sp^3^ bonds are aimed to improve the shear interactions. We find that all these tensile mechanical properties ($\bar{E}$, $\bar{\sigma }$, *Ψ*, and *ε*) are improved with the increased density of sp^3^ bonds from *ρ*_sp3_ = 0.0132 to *ρ*_sp3_ = 0.0395. The same trend can also be observed under shear loading.

### 3.5 Helicoidal‐Inspired Design

**Figure 7:**
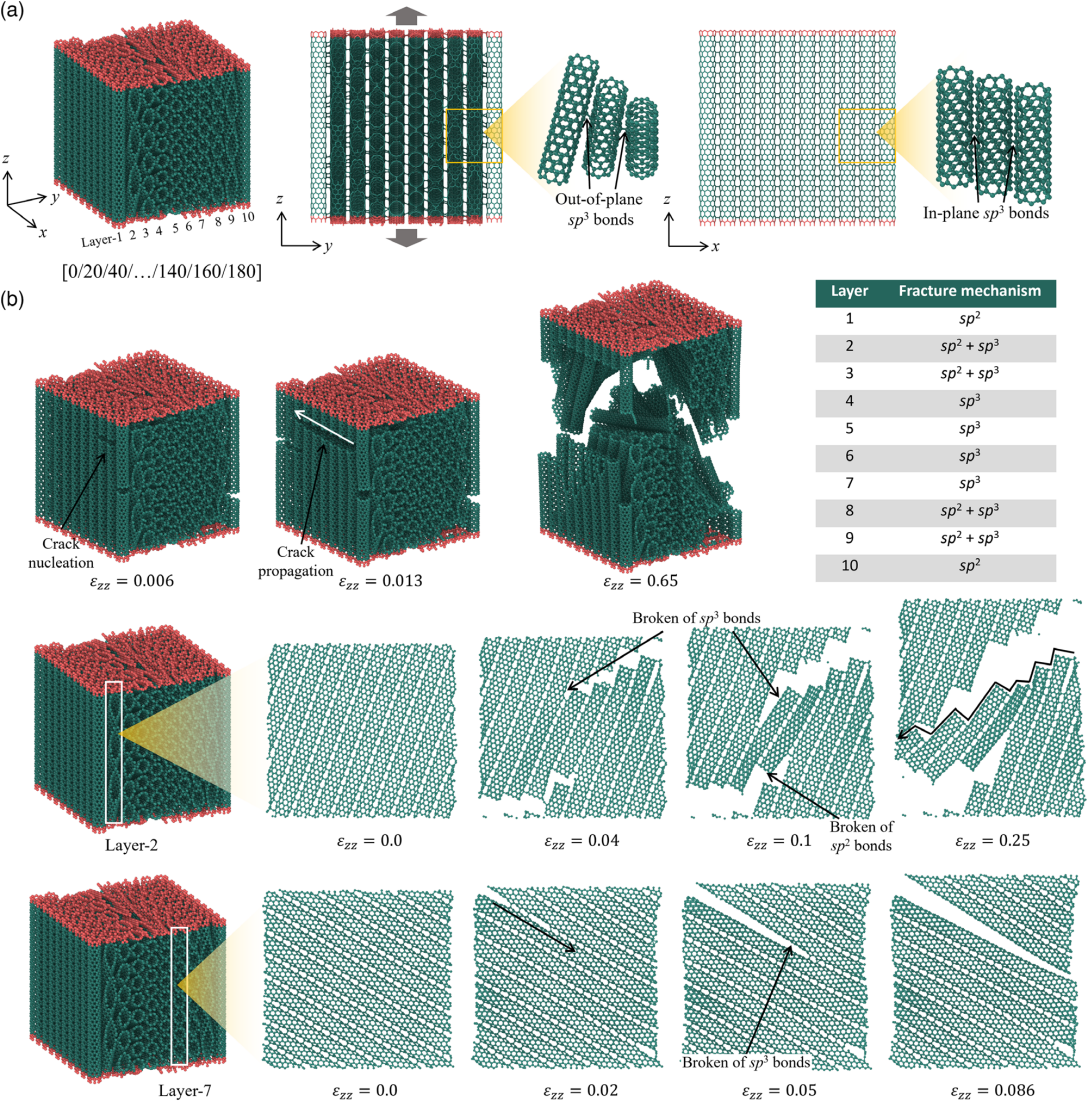
Helicoidal‐inspired CNT metamaterial. a) Schematic of a helicoidal‐inspired CNT metamaterial, its lamination sequence, and sp^3^ bonds connecting CNTs along out‐of‐plane and in‐plane directions. The atoms in red color are fixed for applying tensile displacement in MD simulation, while those in green color are free. b) The atomic configurations and crack propagating process of a helicoidal‐inspired CNT metamaterial. Different fracture mechanisms (breaking of sp^3^ bonds, breaking of sp^2^ bonds, and a mixture of two) at each layer are listed in the graph.

A helicoidal‐inspired metamaterial is composed of ten layers of CNTs, containing ten aligned CNTs in each layer, as shown in **Figure** **7**a. The orientation of CNTs aligned with the *z*‐axis is defined as 0°. A CNT layer is skewed by a 20° angle from the layer next to it, forming a laminating scheme of [0/20/40/…/140/160/180]. The out‐of‐plane sp^3^ bonds are oriented along the *y*‐axis and connect CNTs in the adjacent layers, while the in‐plane sp^3^ bonds link CNTs in the same layer. The densities of the in‐plane sp^3^ bonds and out‐of‐plane sp^3^ bonds are 5.7% and 0.93%, respectively. MD simulation is conducted to study the tensile‐to‐failure mechanical behavior of this metamaterial, and the simulation results are presented in Figure 7b. Cracks emerge at layer‐1 and layer‐10 at a tensile strain of 0.006. During the following deformation, these cracks propagate in the *x*‐direction to break nearly all the CNTs in these two layers. However, these cracks do not bring catastrophic collapse to the metamaterial. With the increased tensile strain, cracks are observed in the middle layers, starting at layer‐2 (*ε* = 0.04) and spreading to layer‐7 (*ε* = 0.086). The cracks can be classified into three types according to the types of the destroyed bonds: sp^2^ crack, sp^3^ crack, and sp^2^ + sp^3^ crack. Pure sp^2^ crack happens at the outer layers, that is, layer‐1 and layer‐10. The fracture mechanism changes into an intermediate state of sp^2^ + sp^3^ crack at layers 2, 3, 8, and 9 and transforms into sp^3^ crack at the middle layers: 5–7. At the tensile strain of 0.086, the helicoidal‐inspired metamaterial shows a sharp reduction of load‐bearing capability, but the energy absorption process continues until *ε* = 0.25. The SEA of helicoidal‐inspired metamaterial is 1.05219 J mm^−3^. As a comparison in the field of continuum mechanics, FEM simulation is conducted with the equivalent method illustrated in Supporting Information S3, Supporting Information, which shows a similar deformation process to the MD results (Figure S3, Supporting Information). Therefore, the toughening mechanisms in both FEM and MD simulations remain the same.

### 3.6 Layered‐Inspired Design

**Figure 8:**
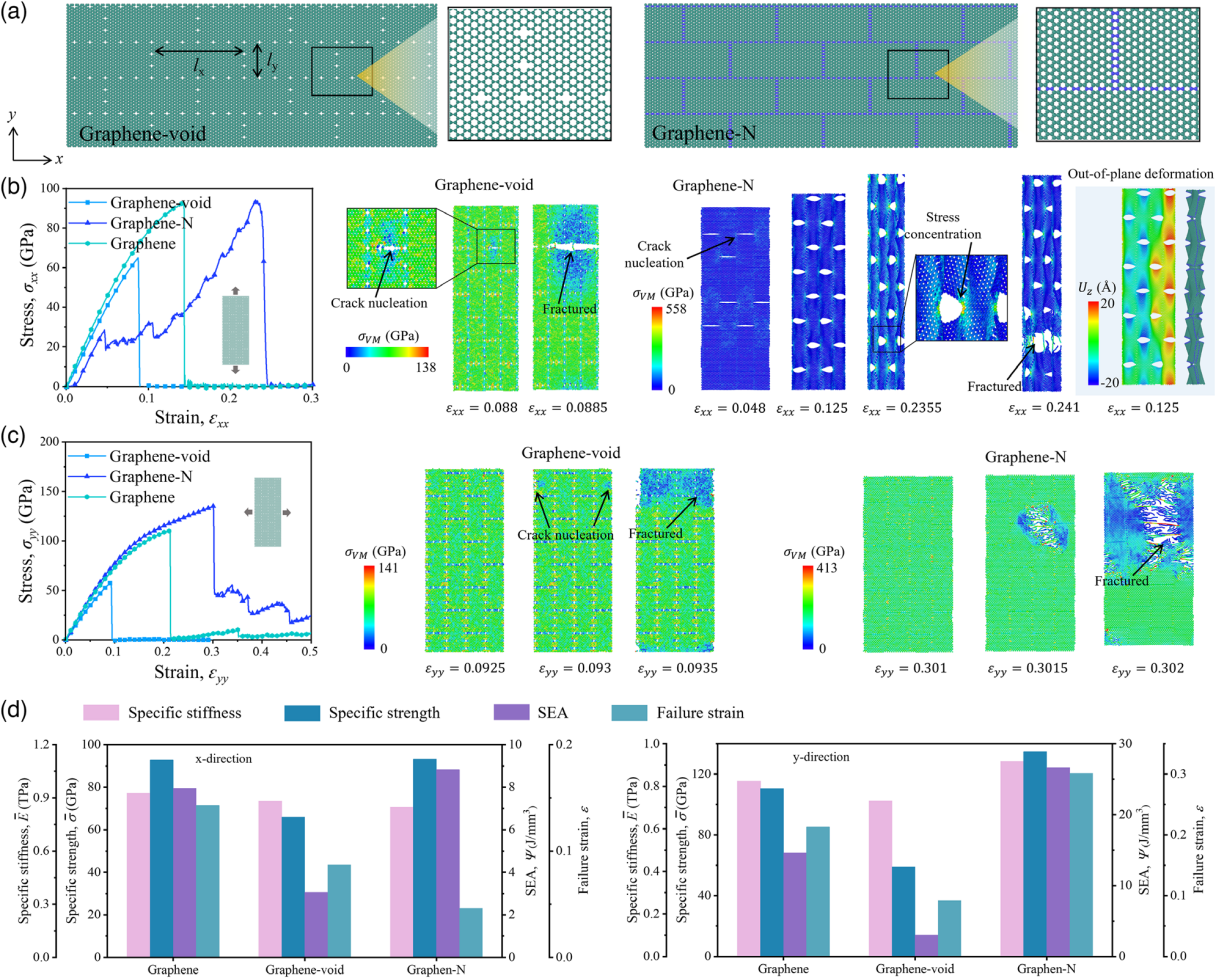
Layered‐inspired graphene metamaterial. a) Schematic of the layered‐inspired graphene metamaterials with brick patterns constructed by different approaches: delating carbon atoms or replacing carbon atoms with nitrogen atoms. b,c) The stress–strain curves of layered‐inspired graphene metamaterials and pristine graphene subjected to tensile load in the *x*‐direction (b) and *y*‐direction (c). The contour displays the von Mises (VM) stress. The right figure in (b) shows the out‐of‐plane deformation of graphene‐N sample with tension in the *x*‐direction, and the contour displays displacement in the out‐of‐plane (*z*‐) direction. d) Mechanical properties (specific stiffness, specific strength, SEA, and failure strain) of layered‐inspired graphene metamaterials and pristine graphene subjected to tensile load in *x*‐ and *y*‐directions, respectively.

Here, we apply the structural element of layered structures into graphene to modify its mechanical properties. **Figure** **8**a depicts the atomic structures and the geometrical parameters (*l*_*x*_ = 75.3132 Å and *l*_*y*_ = 29.5339 Å) of layered‐inspired graphene constructed by two approaches. The first approach is deleting the carbon atoms located in specific locations to from “brick” patterns (graphene‐void). The second one replaces the carbon atoms in specific locations with nitrogen atoms (graphene‐N). One selected area in each design is zoomed in to illustrate the details of brick patterns. Figure 8b,c show the stress–strain curves of pristine graphene and layered‐inspired graphene and illustrate the deformation mechanisms of graphene‐void and graphene‐N metamaterials subjected to the *x*‐ and *y*‐directions, respectively. It is found that the graphene‐void sample shows a similar stress–strain response and deformation mechanism under the tension in two different directions. The deleted atoms result in the stress concentration, leading to the initial crack nucleation (*ε*_*xx*_ = 0.088 and *ε*_*yy*_ = 0.093) and sharp stress decreases shown in stress–strain curves of graphene‐void. The graphene‐N sample exhibits a direction‐dependent stress–strain response and deformation mechanism in Figure 8b,c. When graphene‐N is deformed under the *y*‐direction tensile load, the stress concentration induced crack nucleation and propagation lead to the final brittle fracture. Owing to the distinct brick patterns in the *x*‐ and *y*‐directions, the stress–strain response of graphene‐N in the *x*‐direction can be characterized by three distinct regimes: an initial linear elastic deformation, a plateau stress regime, and a zigzag‐like increased stress regime. The nucleation and propagation of cracks lead to the first stress drop. However, the cracks along the nitrogen lines stop the propagation when reaching the vertical nitrogen lines (*ε*_*xx*_ = 0.048, graphene‐N). At this moment (*ε*_*xx*_ = 0.048), the deformed graphene‐N looks like a graphene sheet with kirigami patterns.^[^ ^65^ ^]^ Then, the following stress–strain response is similar to the post‐buckling response of kirigami sheets, where the plateau stress is induced by the out‐of‐plane deformation of hinges, and the final wavely increasing stress is caused by the stretching of hinges.^[^ 20, 66 ^]^ The out‐of‐plane deformation of graphene‐N (*ε*_*xx*_ = 0.125, graphene‐N) can be observed in the right snapshot in Figure 8b. The mechanical properties of pristine graphene and layered‐inspired graphene metamaterials are summarized in Figure 8d. It is found that graphene‐N shows excellent mechanical properties, especially high specific strength and SEA compared to graphene and graphene‐void. Moreover, the failure strain here is defined as the strain of the first bond broken. The total fracture of graphene‐N occurs at a strain of 0.241, higher than the failure strain of pristine graphene (*ε*_f_ = 0.14).

### 3.7 Suture‐Inspired Design

**Figure 9:**
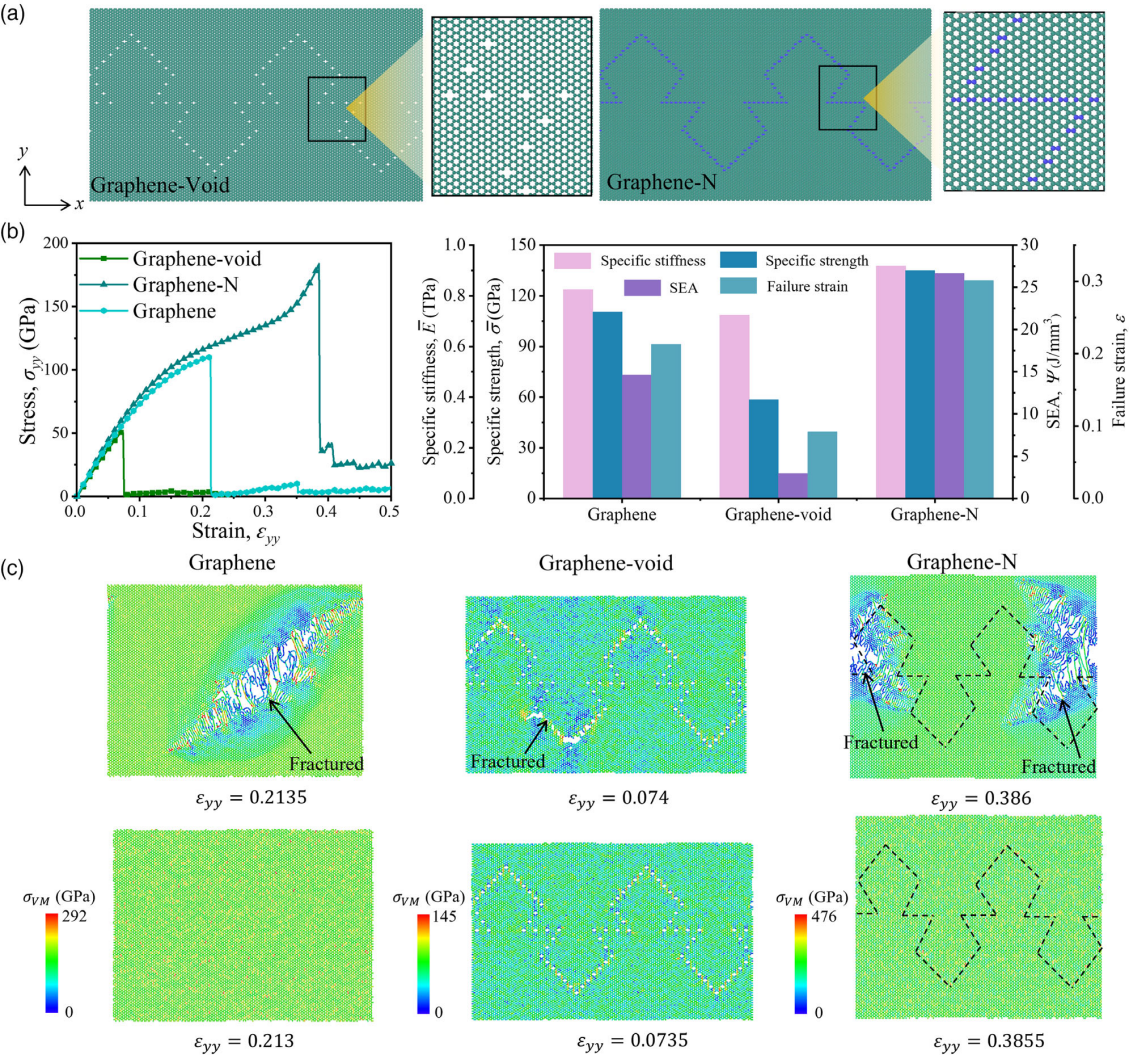
Suture‐inspired graphene metamaterial. a) Schematic of the suture‐inspired graphene metamaterial MD models with interfaces constructed by different approaches: deleting carbon atoms or replacing carbon atoms with nitrogen atoms. b) The stress–strain curves of suture‐inspired graphene metamaterials and pristine graphene subjected to a tensile load in the *y*‐directions. c) Mechanical properties (specific stiffness, specific strength, SEA, and failure strain) of suture‐inspired graphene metamaterials and pristine graphene subjected to tension. d) The deformation‐failure process of suture‐inspired graphene metamaterial in MD simulations. The contour displays the von Mises (VM) stress.

To construct a flexible but strong metamaterial, a suture‐inspired design is introduced. Here, suture nanostructures are constructed by deleting the carbon atoms (graphene‐void) or replacing the carbon atoms with nitrogen atoms (graphene‐N) in graphene sheets, as shown in **Figure** **9**a. As a result, a significant stiffness difference between wavy interfaces and suture teeth is formed. To examine the enhancement of the suture design under uniaxial tensile loading, pristine graphene is selected as the baseline. The stress–strain curves and mechanical properties obtained by MD simulation are summarized in Figure 9b,c. The stress–strain curves of all the models show similar elastic‐brittle trends but vary a lot in terms of failure strain. Compared with the pristine graphene, The graphene‐void design collapse at a lower failure strain (*ε*_*yy*_ = 0.074), which leads to poor strength, stretchability, and energy absorption performances. Among all the models in Figure 9c, graphene‐N shows the highest specific modulus, specific strength, SEA, and failure strain among these designs. The improvement reaches nearly two times in SEA and 81% in failure strain compared with a graphene sheet.

From Figure 9d, the pristine graphene collapses with a straight crack propagating through the material, completely depriving the load‐bearing capability. Even though the graphene‐void changes the propagating path into a wavy one, the stress concentration around the atom vacancy severely reduces the failure strain and other related mechanical properties (specific strength and SEA). The suture‐inspired graphene‐N enhances the graphene by decreasing the stress concentration, dispersing the positions of crack sprouting, and increasing the area of crack bands. As depicted in Figure 9c, compared with the baseline, the graphene‐N shows more crack propagating paths as well as more intricate propagating directions at the failure strain, which greatly hinders the cracks from propagating and therefore enhances the energy dissipation during the crack propagation.

## 4 Discussion

MD and FEM approaches are applied to study the mechanical properties and deformation of cellular‐ and gradient‐inspired designs. Similar conclusions about the stiffness can be drawn from MD and FEM results. For example, in both approaches, the hierarchical designs realize the improvement of specific stiffness compared with the nonhierarchical one, and the improvement is more significant as the hierarchical level increases. Failure‐related properties, including specific strength, failure strain, and SEA, depend on the definition of failure criterion and modeling geometries. In MD simulation, the cutoff distance between carbon and carbon bonds is utilized to define the failure of graphene and graphene‐based metamaterials. While in FEM, the maximum failure strain criterion is applied to capture the failure of the designs. The modeling geometries are another critical factor resulting in the difference between MD and FEM results regarding the failure‐related properties. As shown in Figure 3a, a detailed geometry containing the position information of every atom is utilized in MD. Due to lack of accurate mechanical parameters of the carbon sp^2^ bonds, the graphene sheet with atoms arranged in the 2D honeycomb lattice is equivalent to a homogenized solid plate in FEM, which brings negligible deviation on the prediction of stiffness (Figure S4, Supporting Information) but affects the failure strain significantly. Hence, a detailed FEM model with beam elements mimicking the carbon sp^2^ bonds is needed in further study, where a modified beam theory could be applied to accurately predict the failure behaviors of carbon sp^2^ bonds.

A wide range of combinations of mechanical merits (specific stiffness, strength, failure strain, and SEA) can be obtained by tailoring the geometric configuration of the bioinspired metamaterials. Here, we provide a case study on the effect of stretching‐/bending‐ dominated configurations. Results show that the stretchability, namely failure strain, obtains the most significant improvement, among the four mechanical properties examined in this study. For example, the 1st order cellular‐inspired graphene metamaterial increases the stretchability of graphene by ≈100%. Simultaneously, the specific strength is increased by a smaller magnitude. Furthermore, the stiffness of cellular‐inspired metamaterials can be increased at the cost of stretchability if we apply the stretch‐dominated architectures instead of the hexagonal configuration as building elements. One good example is using FCC as stretch‐dominated architecture for designing metamaterials.^[^ ^67^ ^]^ Graphene‐based FCC^[^ ^32^ ^]^ and CNT‐based FCC^[^ ^21^ ^]^ metamaterials exhibit much higher strength than the cellular‐inspired designs in this study (the strengths of the graphene FCC and CNT FCC are around 8 and 10 GPa). However, their stretchability is very limited; the failure strain of graphene FCC and CNT FCC metamaterials is below 0.1 and 0.2, separately. Moreover, the crack nucleation and propagation path in carbon‐based metamaterials can be manipulated to increase the stretchability and change the failure modes. For example, the ordered distributed tubular in tubular‐inspired designs make the crack nucleation uniformly. By introducing layered‐inspired configuration to the graphene‐based metamaterials, a ductile failure process with a long‐time crack propagation and a high resultant SEA can be acquired. This phenomenon is similar to the post‐buckling response of kirigami structures.

**Figure 10:**
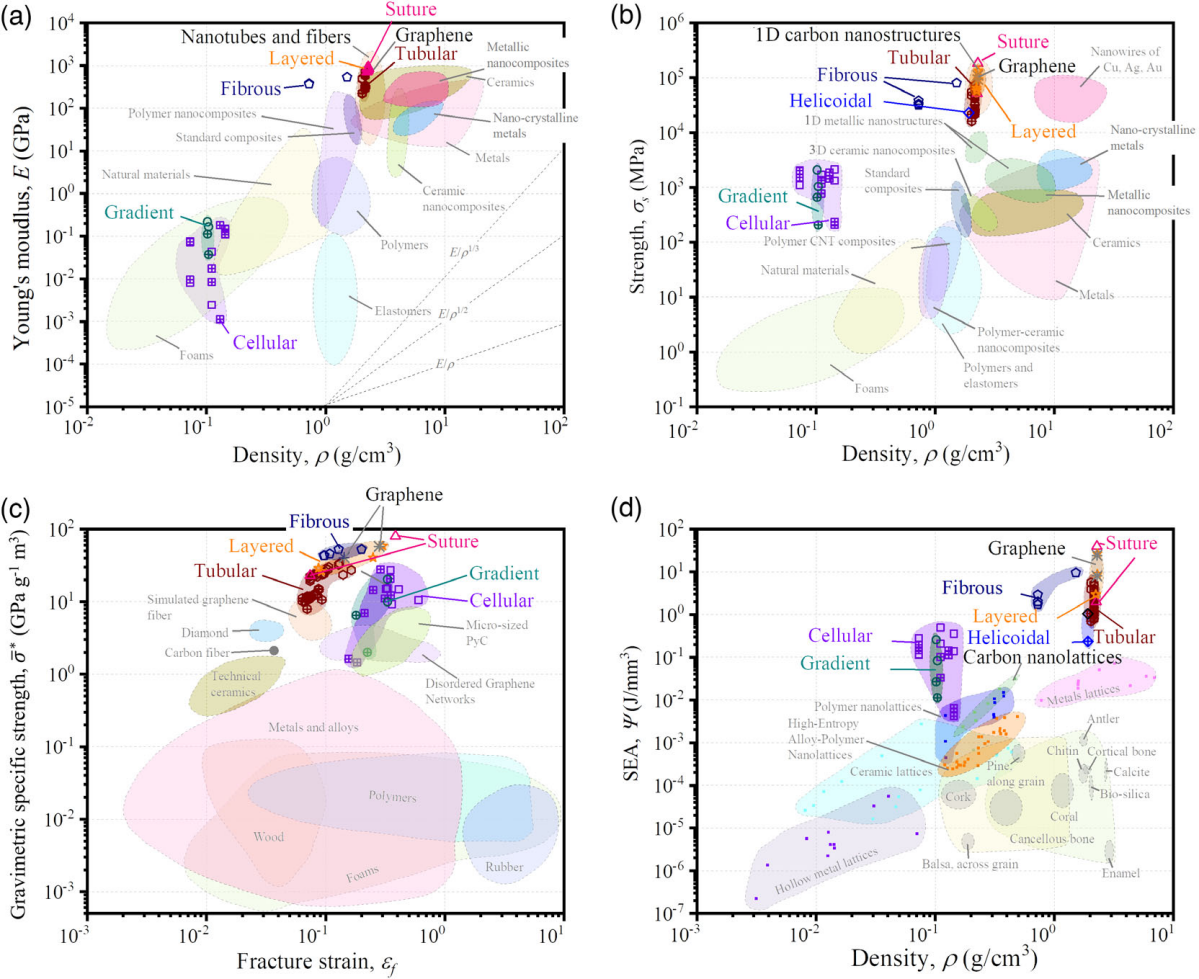
Material selecting guide for carbon‐based nanoarchitected mechanical metamaterials. For the sake of comparison, the properties of common natural and synthetic materials are also presented. Ashby charts for: a) Young's modulus (*E*) versus density; b) tensile strength ($\sigma_{s}$) versus density; c) gravimetric specific strength (${\overset{¯}{\sigma }}^{*}$) versus fracture strain ($\varepsilon_{f}$); d) SEA (*Ψ*) versus density; some cellular materials with high energy absorption capabilities are shown here for comparison. The data are from the literature.^[^ ^85^ ^]^

The material property charts (also named Ashby charts) in **Figure** **10**a,b show Young's modulus (*E*) and tensile strength (*σ*_s_) versus density for a wide range of nanoscale carbon‐based materials, other nanomaterials, as well as commercially available materials. The base materials of the introduced bioinspired nanoarchitected metamaterials, i.e., graphene and nanotube, are also indicated in the charts. Compared to the conventional nanomaterials and commercial materials,^[^ ^68^ ^]^ the proposed bioinspired carbon‐based metamaterials, except for cellular‐ and gradient‐inspired designs, show higher stiffness and ultimate tensile strength, filling the upper‐left empty regions in Ashby charts (Figure 10a,b). The stiffness and strength of the lightweight tubular‐, fibrous‐, layered‐, suture‐, and helicoidal‐inspired carbon‐based metamaterials are comparable to those of carbon fibers, metallic nanocomposites, and nanowires of metals,^[^ ^69^ ^]^ while their densities are only 8%–20% of the metallic nanowires. Cellular‐ and gradient‐inspired graphene metamaterials achieve comparable stiffness and two orders of magnitude higher strength compared to conventional foams. The ultimate strengths of cellular‐ and gradient‐inspired samples are around 1000 MPa with a relatively low density (ρ= 0.1 g cm^−3^). Figure 10c shows the Ashby plot for specific strength versus failure strain for the proposed bioinspired carbon‐based metamaterials along with a variety of other materials, including commercially available bulk materials, graphene, carbon fibers,^[^ ^70^ ^]^ micro‐sized pyrolytic carbon,^[^ 71, 72 ^]^ disordered carbon networks,^[^ ^73^ ^]^ and polycrystalline diamond.^[^ ^74^ ^]^ The specific strength of tubular‐, fibrous‐, layered‐, and suture‐inspired carbon‐based metamaterials outperform all other materials displayed in the chart, occupying a hitherto unexplored space in the Ashby diagram. It is worth mentioning that the failure strain of the carbon‐based metamaterials in this work is defined as the strain at which the first carbon bond is broken. Hence, the fracture strain associated with the complete failure of the metamaterials is greater than the reported failure strain in Figure 10c, owing to the ductile fracture behavior induced by the bioinspired designs. For instance, the failure strains of 2nd order cellular‐ and layered‐inspired (graphene‐N) graphene metamaterials are 0.312 and 0.046, while the fracture strains are around 0.6 and 0.25 for these two designs. In this viewpoint, cellular‐ and layered‐inspired graphene metamaterials could overcome the classical trade‐off between strength and ductility in materials science. Figure 10d presents the landscape for SEA versus density for the carbon‐based metamaterials and alternative engineered lattice materials (e.g., metal, polymer, and ceramic lattice) and natural materials (e.g., pine, antler, and bone). Particularly, all bioinspired carbon‐based metamaterials exhibit SEA values that surpass almost all other materials shown here. For instance, cellular‐inspired graphene metamaterials achieve at least one order of magnitude greater SEA than the metal lattices, while the density of the cellular‐inspired graphene metamaterials is less than 10% of the metallic lattice counterparts. It is worthwhile mentioning that the effect of tailoring geometrical and topological parameters on the performance of nanoarchitected materials has not been studied here, requiring further studies on topological optimization of their nano/microarchitecture (see Supporting Information S6, Supporting Information).

Except for CNT and graphene, the library of constructing building blocks for bioinspired nanoarchitected metamaterials can be expanded to achieve a wider multifunctional properties. For instance, the covalent networks of carbon and nitrogen atoms produce various 2D carbon‐nitride nanostructures, e.g., C_3_N,^[^ ^75^ ^]^ C_3_N_4_,^[^ 76, 77 ^]^ and C_2_N,^[^ ^78^ ^]^ as building blocks for developing nanoarchitected metamaterials, as shown in Figure S14, Supporting Information. The mechanical response of C_2_N‐ and C_3_N‐based cellular‐inspired metamaterials is examined through MD simulation. The stress–strain curves, as shown in Figure S14b(i), Supporting Information, present the outstanding stretchability of the abovementioned designs.

Apart from mechanical responses, it is worth noting that the 2D base nanomaterials also show other multiphysical properties. For example, the pristine monolayer graphene shows ultrahigh thermal conductivity (3000–5000 W m^−1^ K^−1^^[^ 8, 12 ^]^). Here, we take the tubular‐inspired graphene metamaterials as an example to study the thermal transport in bioinspired metamaterials in Figure S14b(ii), Supporting Information. The thermal conductivities of tubular samples (*κ* = 60.61 W m^−1^ K^−1^, and 60.92 W m^−1^ K^−1^) decrease significantly compared to the pristine graphene (*κ* = 225.59 W m^−1^ K^−1^), as shown in Figure S14b(ii), Supporting Information. Hence, the combination of bioinspiration illustrated in this work and the unique properties of 2D base materials could bring some unexpected multiphysical properties and multifunctionality, which can broaden their potential applications.

## 5 Concluding Remarks

We have designed a series of carbon‐based nanoarchitected metamaterials by inspiring from the seven biomaterial motifs and demonstrate that the bioinspired carbon‐based metamaterials possess enhanced mechanical properties, occupying hitherto unexplored spaces in the material property chart and imparting possibilities to break the performance trade‐offs found in conventional materials. Utilizing graphene and CNT as building blocks, seven classes (i.e., cellular, gradient, tubular, fibrous, helicoidal, layered, and suture structures) of bioinspired structural‐design elements are developed to construct nanoarchitected metamaterials. Two simulation methods (MD and FEM) are resorted to explore their mechanical properties and disclose their deformation mechanisms. The computational results indicate that the nanoarchitected designs based on bioinspiring motifs enrich, and in some cases improve, the mechanical responses of graphene and CNT nanomaterials. While a single structural‐design element has been used in the current study to develop nanoarchitected synthetic materials, biological materials in nature often exhibit hybrid structural elements. The combination of these structural‐design elements for designing nanoarchitected metamaterials is deemed to bring further enhancement to their multifunctional properties. The synchronous involvement of bioinspired design strategies in this work and other 1D/2D nanomaterials with unique multiphysical properties can open avenues for designing new high‐performance nanoarchitected metamaterials with programmable thermo‐electro‐mechanical properties for applications in smart nanorobots,^[^ ^79^ ^]^ reconfigurable nanocapsules,^[^ ^80^ ^]^ nanogenerators,^[^ ^81^ ^]^ and protective nanomaterials.^[^ ^23^ ^]^

## Conflict of Interest

The authors declare no conflict of interest.

## Supporting information

Supplementary Material
