# Supplementary material for: Lessons from Nature for Carbon‐Based Nanoarchitected Metamaterials
Source: Small Sci. 2022 Nov 13;2(12):2200039. doi: 10.1002/smsc.202200039 (PMC11935877; doi:10.1002/smsc.202200039)
Supplement: Supplementary file 1 — Supplementary Material [file SMSC-2-2200039-s001.pdf]

Supporting Information

Lessons from nature for carbon-based nano-architected metamaterials

Jun Cai [†], *Haoyu Chen* [†], *Youjian Li, Abdolhamid Akbarzadeh* *

S1. Calculation of the volume of designed metamaterials

S2. FEM results of tubular-inspired metamaterials

S3. FEM results of helicoidal-inspired metamaterials

S4. Validation of finite element simulation

S5. Relative density

S6. Geometric parameters of designed metamaterials

S7. Stress-strain curves of pristine graphene

S8. Designs of Pomelo peel-inspired graphene metamaterials

S9. MD results of cellular-inspired metamaterials with periodic boundary conditions

S10. Effect of the rows on the mechanical properties of gradient-inspired designs

S11. Designs of SSD and RSD samples

S12. MD results of tubular-inspired metamaterials

S13. Relaxation of CNT bundles

S14. Potential candidates of low-dimensional nanomaterials

---

* Corresponding author: hamid.akbarzadeh@mcgill.ca

[†] Jun Cai and Haoyu Chen contributed equally to the work.

S1. Calculation of the volume of designed metamaterials

When calculating the stress (Equation 1 in the main text) and the relative density, the volume (V0) of the bioinspired nano-architected metamaterials before applying any deformation is an important parameter. In Figure S1, we select three representative samples to illustrate the definition of V0 in this work. The volume is calculated as $V_0 = l_x \times l_y \times l_z$.

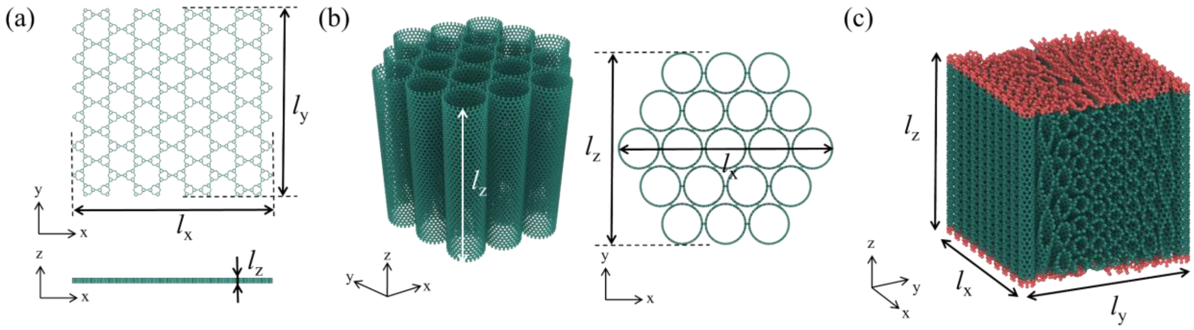

Figure S1. The geometric parameters of (a) 3rd order cellular-, (b) fibrous-, and helicoidal-inspired carbon-based metamaterials.

S2. FEM results of tubular-inspired metamaterials

The geometries of tubular-inspired FEM models are based on the corresponding MD models. Translational ($\overleftrightarrow{z}$) and rotational ($\widehat{xz}$ and $\widehat{yz}$) degrees of freedom (DoFs) are constrained for all the beam elements to ensure the plane strain status in the *x*-*y* plane. All the DoFs are constrained for the nodes at one edge of the model, while only the translational DoF along the loading direction is released at the opposite edge, where a prescribed motion with uniform speed is applied. In the meanwhile, symmetric boundary conditions are set at the other two edges. The volumes of carbon atoms and sp^2 bonds are converted into cylindrical strut members of a porous material. The positions of connecting nodes of the struts are inherited from the carbon atom positions in MD models. The effective diameter $\tilde{r}$ of the struts is defined by the following equation:

$$\tilde{r} = \sqrt{\frac{n_a \cdot V_{Carbon}}{l_s \cdot \pi n_s}} \tag{S1}$$

where n_a, n_s, V_{Carbon}, l_s are the number of carbon atoms in a volume of graphene in the MD model, the number of struts in a same volume of equavalent FEM model, the volume of a carbon atom in the graphene, and the length of a strut, respectively. Based on the effective thickness (1.27 Å) applied in FE calculation, the equivalent diameter of sp^2 bonds used in FE analysis is 1.41 Å. As shown in Figure S2, the FEM results also present a elastic-brittle failure process, which is consistent with the phenomena observed in MD results.

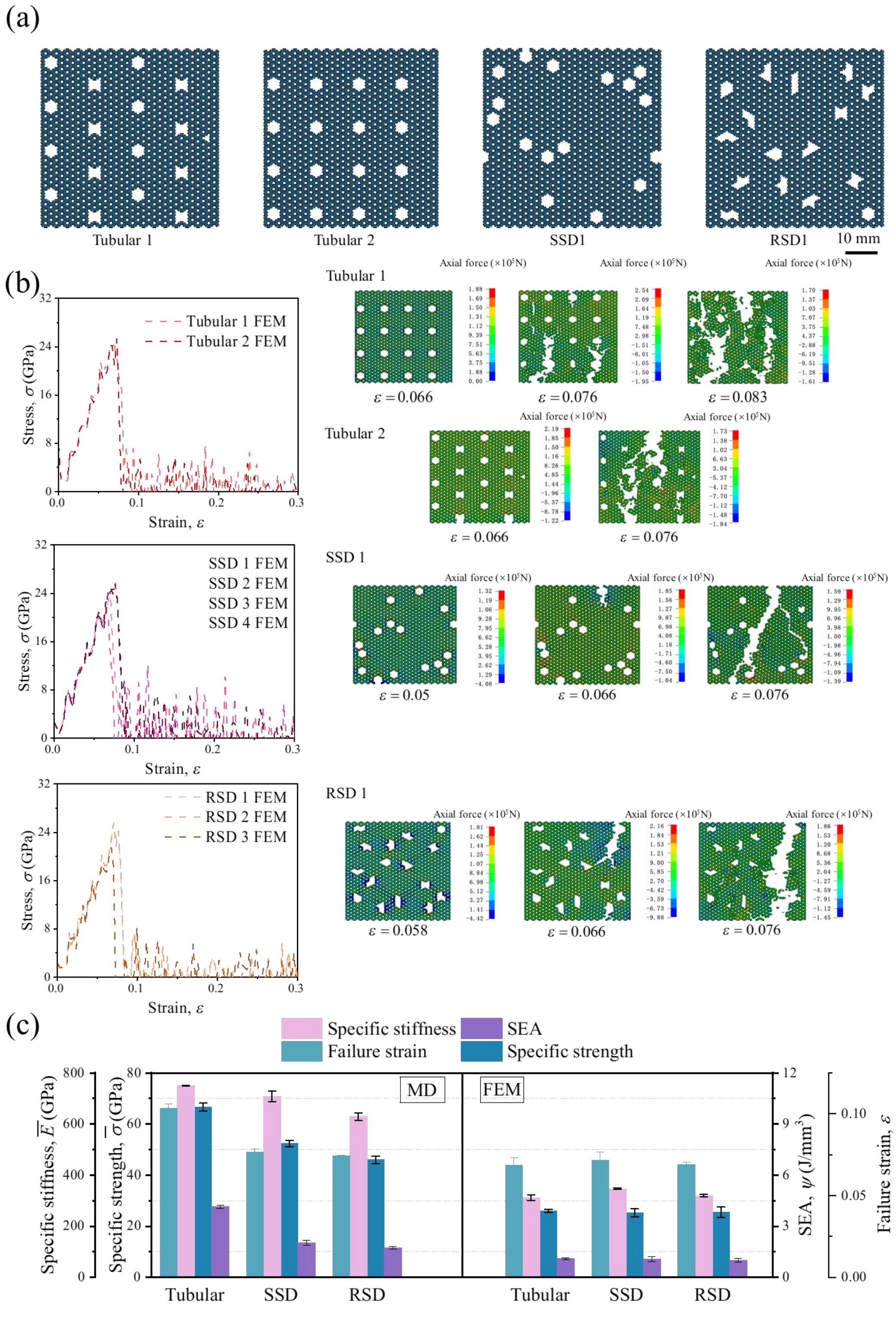

Figure S2. FEM results of tubular-inspired graphene metamaterial. (a) Schematic of FEM model of Tubular 1, Tubular 2, SSD1, and RSD1. (b) Stress-strain curves of tubular-inspired graphene metamaterials and samples in SSD and RSD group under a tensile load in the x-direction obtained by FEM simulations, and the corresponding damage status at certain tensile strains. (c) Mechanical properties (specific stiffness, specific strength, SEA, and failure strain) of tubular-inspired graphene metamaterials, SSD, and RSD obtained by MD and FEM simulations.

S3. FEM results of helicoidal-inspired metamaterials

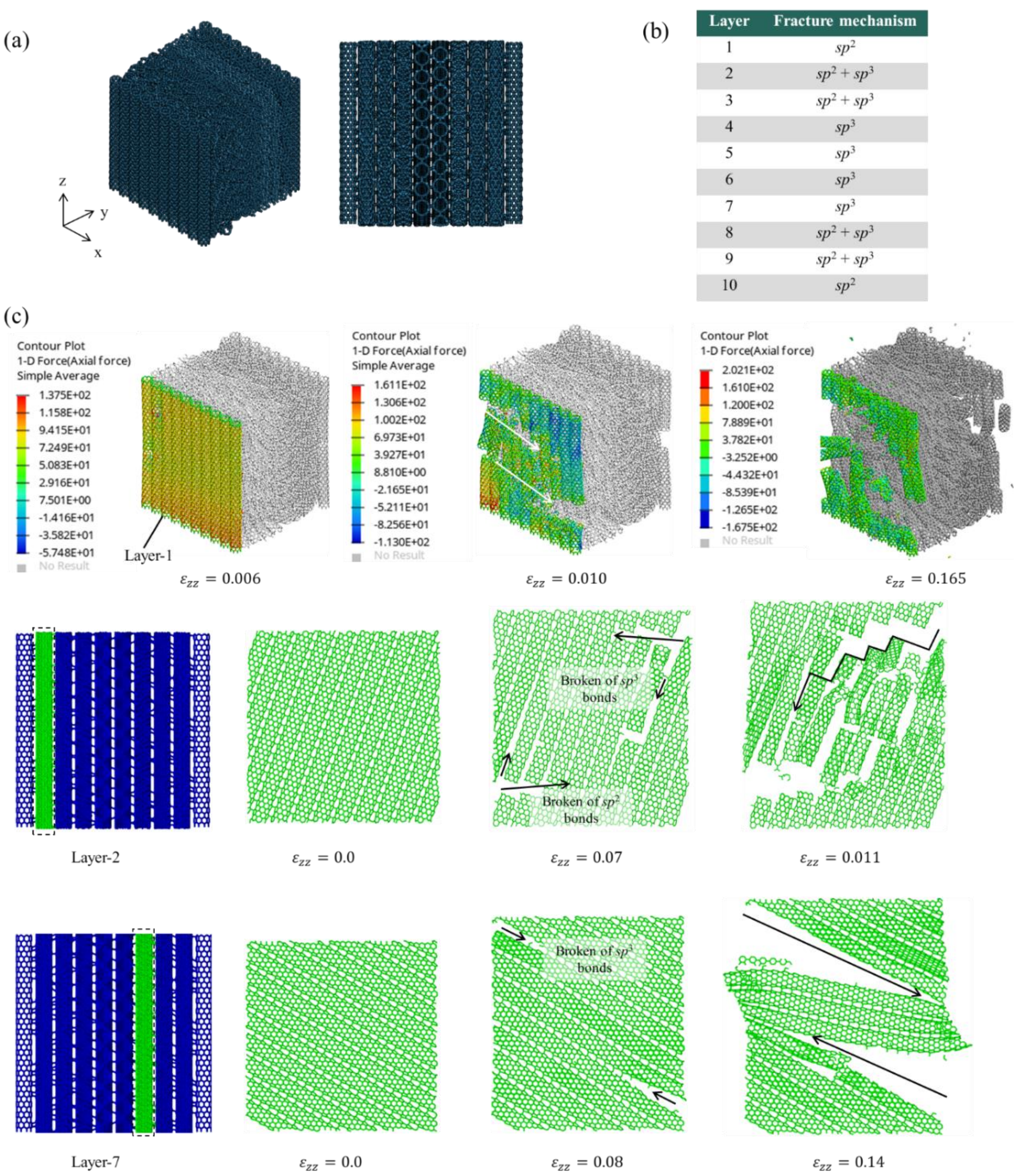

Layer	Fracture mechanism
1	sp^2
2	$sp^2 + sp^3$
3	$sp^2 + sp^3$
4	sp^3
5	sp^3
6	sp^3
7	sp^3
8	$sp^2 + sp^3$
9	$sp^2 + sp^3$
10	sp^2

Figure S3. FEM simulation results of helicoidal-inspired CNT metamaterial. (a) FEM model of a helicoidal-inspired CNT metamaterial: isotropic view and sectional view (b) Different fracture mechanisms (breaking of sp^3 bonds, breaking of sp^2 bonds, and a mixture of two) at each layer. (c) The simulated deformation and crack propagating process of layer 1, 2, and 7 in a helicoidal-inspired CNT metamaterial.

S4. Validation of finite element simulation

Figure S4 shows the effect of the cell length (c) on the tensile stiffness of 1st order cellular-inspired graphene metamaterials. At the continuum level, the tensile stiffness of a honeycomb structure is predicted to be $E \propto (t/c)^3 E_0$, where E and E_0 are the tensile stiffness of the honeycomb structure and the base material, respectively. t and c are the thickness of the base material and the cell length of the honeycomb structure, respectively. As shown in Figure S3, our MD and FE results can be well fitted by the theoretical predictions.

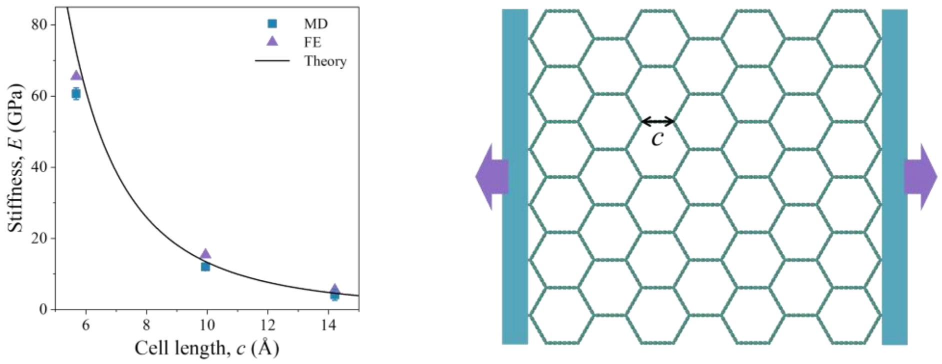

Figure S4. The tensile stiffness of 1st order cellular-inspired graphene metamaterials as a function of the cell length.

S5. Relative density

Table S1. Relative densities of proposed bioinspired carbon-based nano-architected metamaterials

Designs	Cellular			Tubular	Gradient	
	H1	H2	H3		G1	G2
$\bar{\rho}$	0.3197	0.8428	0.9815	1.0	0.9919	1.0

Designs	Fibrous	Helicoidal	Layered		Suture	
			Graphene-void	Graphene-N	Graphene-void	Graphene-N
$\bar{\rho}$	0.3197	0.8428	0.9815	1.0	0.9919	1.0

S6. Geometric parameters of designed metamaterials

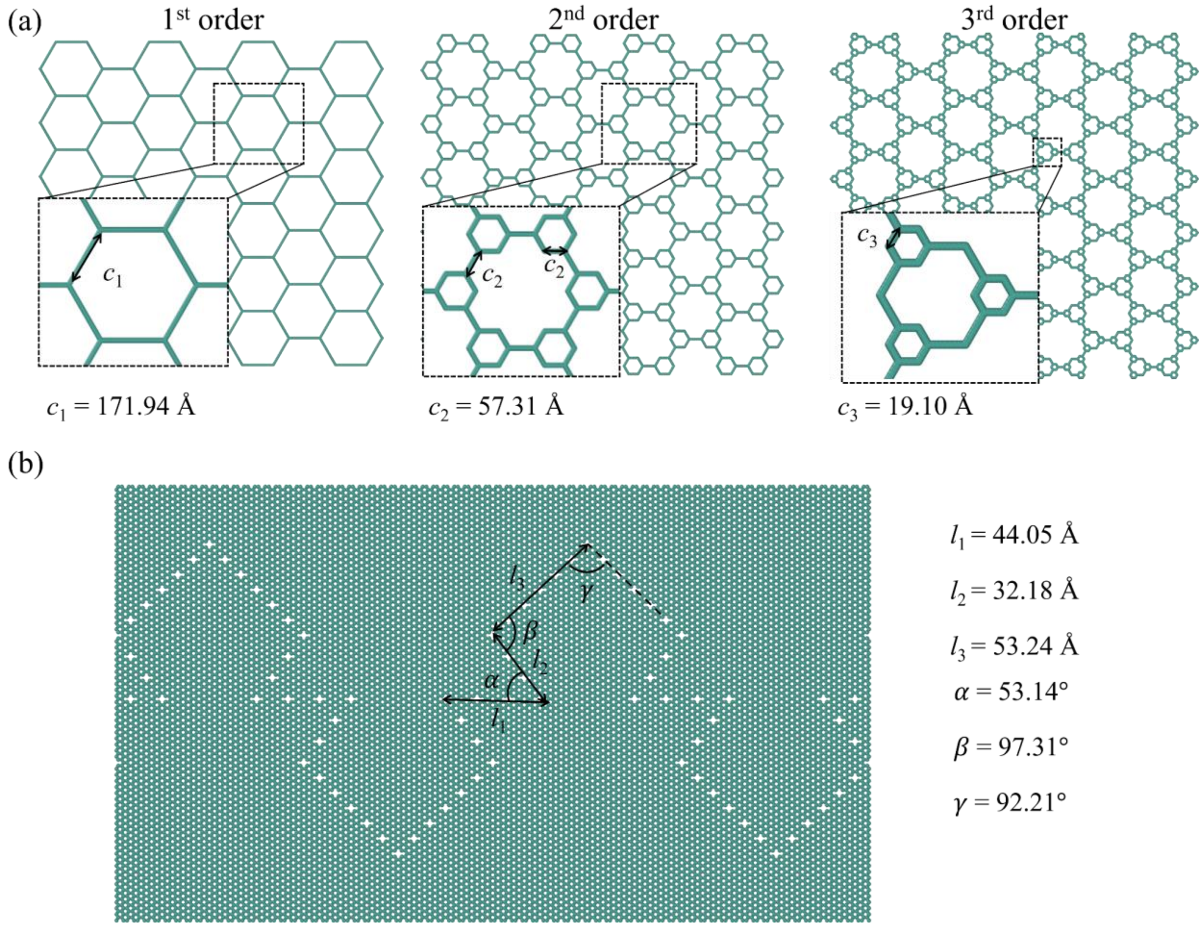

Figure S5. The geometric parameters of (a) cellular-inspired and (b) suture-inspired graphene metamaterials.

S7. Stress-strain curves of pristine graphene

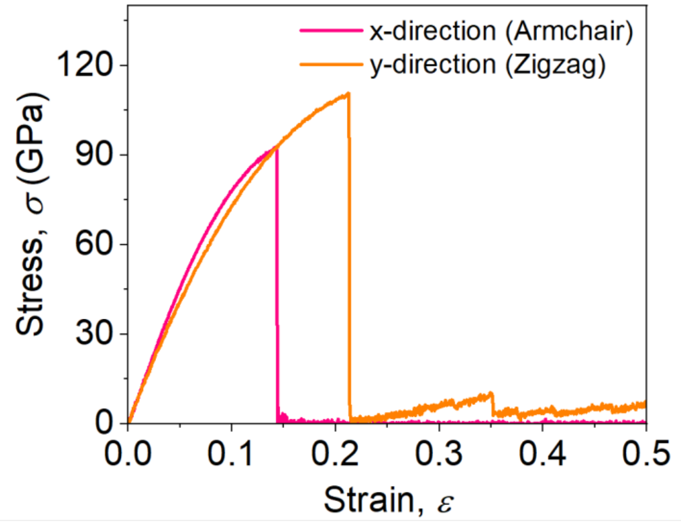

Figure S6. Stress-strain curves of pristine graphene subjected to the tensile along the x- (Armchair) and y-direction (Zigzag). The periodic boundary conditions are applied to the in-plane directions. AIREBO [1] potential function is used to describe C-C interatomic interactions.

S8. Designs of pomelo peel-inspired graphene metamaterials

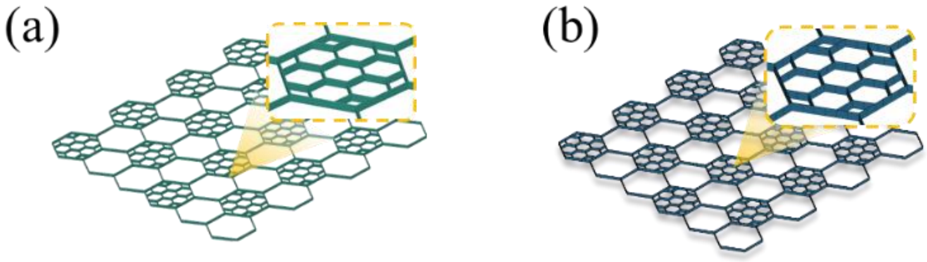

Figure S7. Pomelo peel-inspired graphene metamaterials. (a) MD model; (b) FEM model. The micro-structure of pomelo peels was revealed in a previous literature [2].

S9. MD results of cellular-inspired metamaterials with periodic boundary conditions

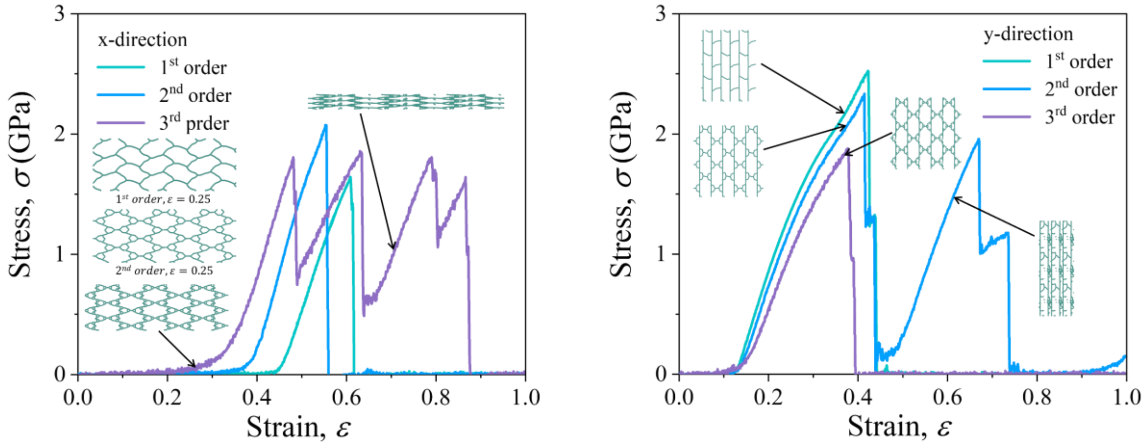

Figure S8. Stress-strain curves of cellular-inspired graphene metamaterials. The periodic boundary conditions are applied in the *x*-, *y*-, and *z*-directions.

S10. Effect of the rows on the mechanical responses of gradient-inspired designs.

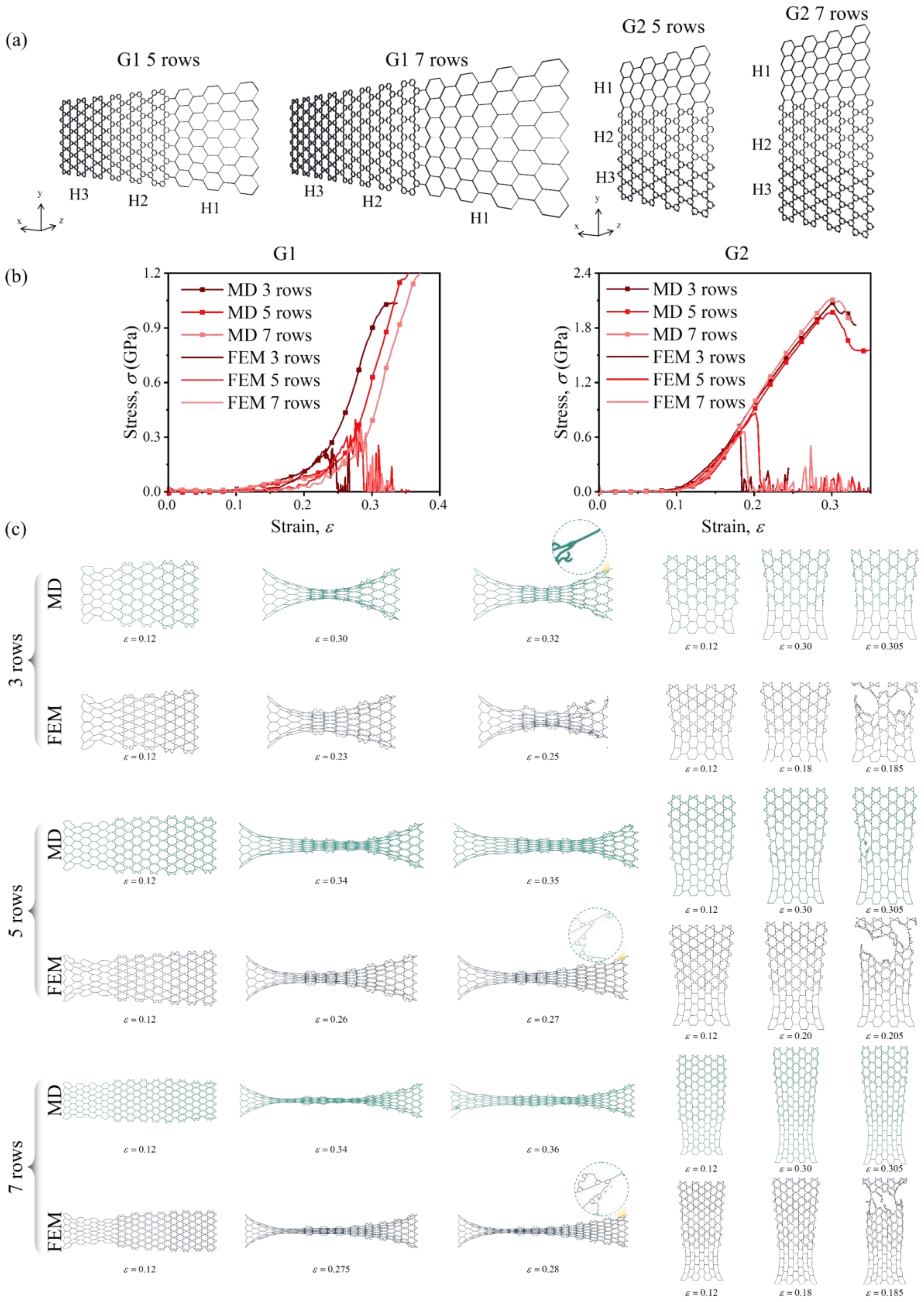

Figure S9. (a) Schematic of gradient designs with 5 rows and 7 rows in G1 (*x*-direction) and G2 (*y*-direction). ((b) Stress-strain curves of gradient-inspired graphene metamaterials under a tensile load along the gradient orientations obtained by MD and FE simulations, and (c) the corresponding configurations at certain tensile strains.

S11. Designs of SSD and RSD samples

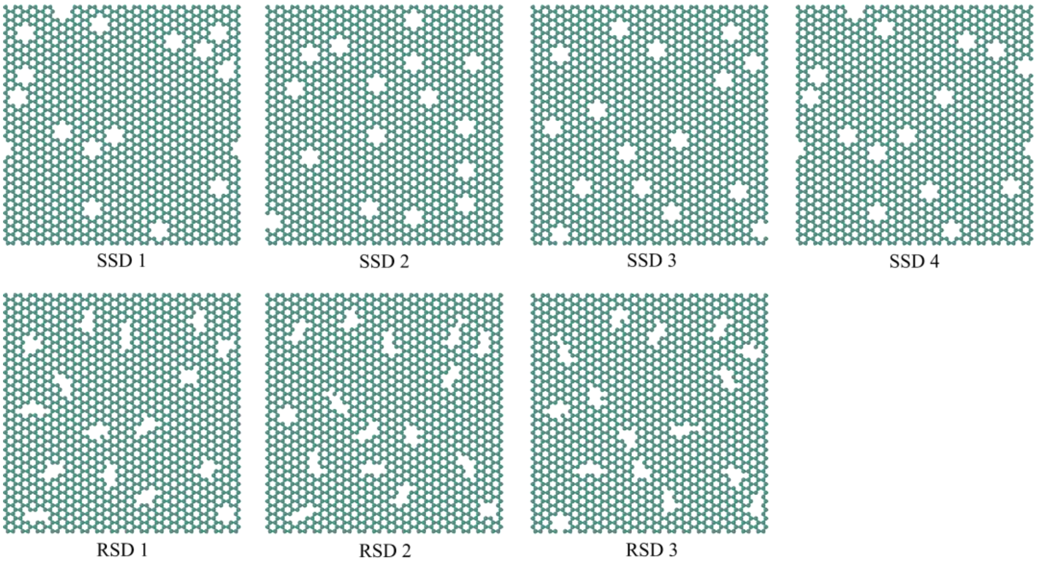

Figure S10. Schematic of tubular-inspired graphene metamaterials (Tubular 1 and Tubular 2) and the graphene sheets with standard shape distribution (SSD) and with random shape distribution (RSD) constructed in MD simulation.

S12. MD results of tubular-inspired metamaterials

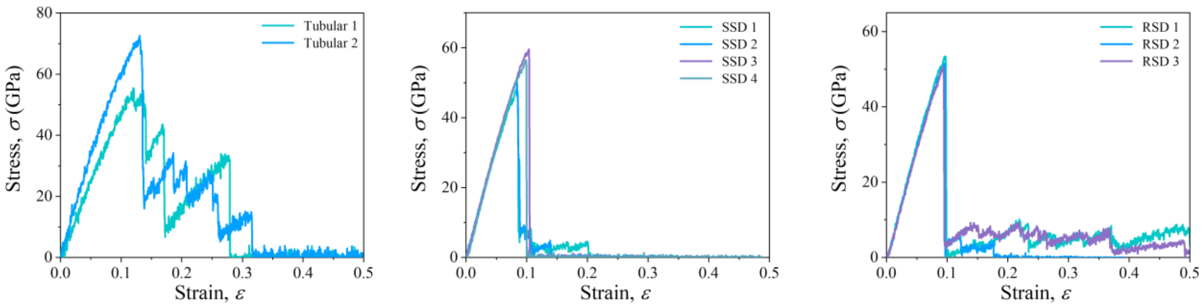

Figure S11. Stress-strain curves of tubular-inspired graphene metamaterials and samples in SSD and RSD group under a tensile load in the *y*-direction obtained by MD simulations. The tensile stress-strain responses in the *y*-direction are similar to the tenisle stress-strain responses in the *x*-direction.

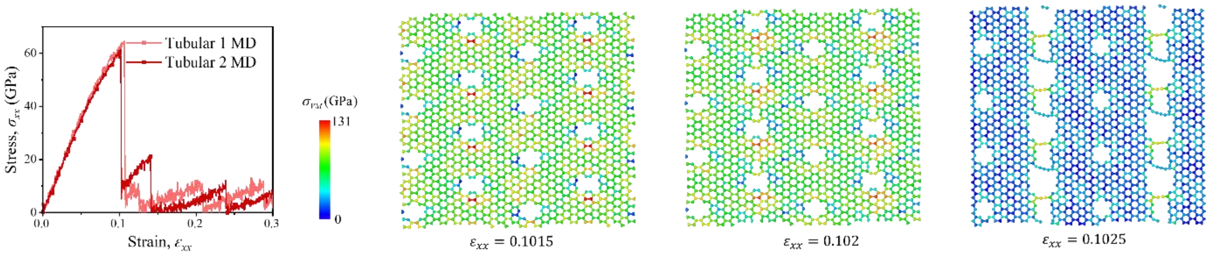

Figure S12. Stress-strain curves of tubular-inspired graphene metamaterials under a tensile load in the *x*-direction obtained by MD, and the configurations of Tubular 2 at certain tensile strains. The contour indicates the von Mises stress.

S13. Relaxation of CNT bundles

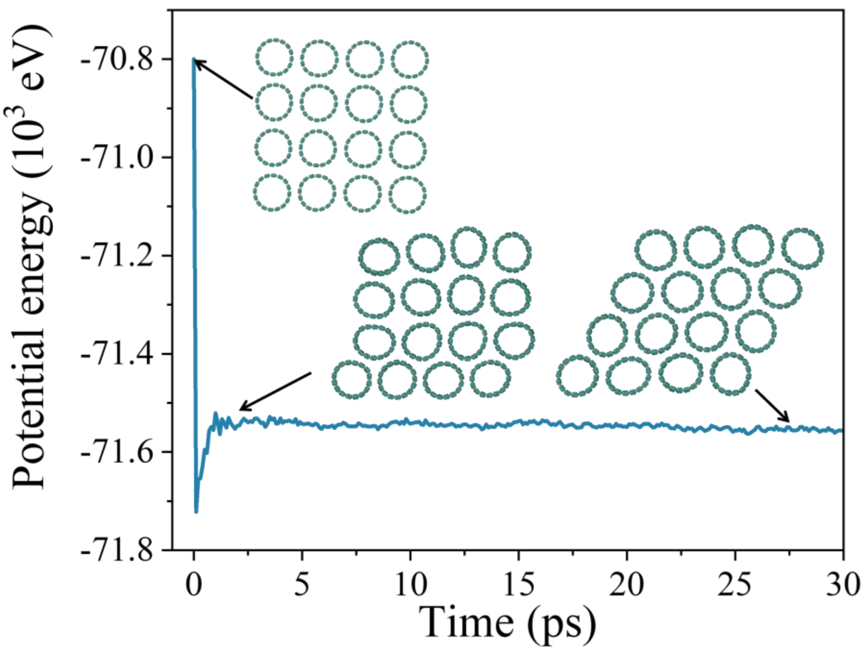

Figure S13. The potential energy of fibrous-inspired CNT bundle as a function of relation time and the corresponding atomic configuration at different relaxation time. It is found that the square CNT bundle is transfered to a triangular lattice after the process of energy minimization and fully relaxation.

S14. Potential candidates of low-dimensional nanomaterials

The structure of C_2N was first fabricated, and its energy bandgap was determined in 2015 [3]. C_3N and C_3N_4 were also successfully synthesized with semiconducting properties in the Laboratories [4, 5]. Graphdiyne (GDY) family, formed by the combination of *sp*- and *sp*^{2}-hybridized carbon atoms according to a certain periodicity rule [6], also shows properties distinctive from pristine graphene and CNT, owning to the unique pore structures, and can be exploited to form nano-architected metamaterials.

Here, we show two examples that utilize the aforementioned 2D materials (C_2N and C_3N) to construct the regular honeycomb nano-architected metamaterials with the design ideal illustrated in Section 3.1 (Figure S14b). The cell length of the honeycomb cell is 14.21 Å. The simulation temperature is set to 1 K in order to eliminate undesired thermal effects [7] and stabilize the designed nanostructure. The stress-strain curves of 1st honeycomb nanostructures constructed by graphene, C_2N, and C_3N suggest that the mechanical properties can also be tuned by based materials (Figure S14b (i)). For instance, compared to 1st honeycomb graphene, the strength is increased in 1st honeycomb C_3N metamaterial and the stretchability is improved in 1st honeycomb C_2N metamaterial. In the main text, the mechanical properties of bioinspired carbon-based nano-architected metamaterials are obtained with a simulation temperature of 300 K and a strain rate at 1×10^9 s^{-1}. Previous studies reveals that the mechanical properties of nanoscale graphene/CNT or graphene/CNT-based metamaterials are temperature and strain rate dependent [8-11]. The effect of temperature and strain rate, as well as the geometrical parameters, on the mechanical properties of bioinspired carbon-based nano-architected metamaterials are not explored in this study. For example, we find that the stiffness of regular honeycomb graphene metamaterials is affected by the cellular length (c) and is predicted to be $E \propto (t/c)^3 E_0$, where E and E_0 are the stiffness of regular honeycomb graphene metamaterials, respectively, and t is the thickness of graphene sheet (see Supporting Information S4). Hence, a detailed study exploring the effect of temperature, strain rate, and geometrical parameters on the mechanical properties of bioinspired nano-architected metamaterials can be conducted in the future.

To assess the combination of bioinspiration and the unique multi-physical properties of 2D base materials, we take the tubular-inspired graphene metamaterials as an example to study the thermal transport in bioinspired metamaterials in Figure S14b (ii). In tubular-inspired graphene metamaterials, the ordered distributed voids are aimed to decrease the stress concentration compared to graphene with randomly distributed voids. While in heat conduction, the voids

hinder the temperature transport (see the temperature distributions in Figure S14b (ii)) and scatter the acoustic phonons (see the density of states (DOS) of graphene and fibrous-inspired graphene metamaterials in Figure S14b (ii)), which dominate the heat condition in carbon materials [12-14]. As a result, the thermal conductivities of tubular samples (κ = 60.61 W/mK, and 60.92 W/mK) decrease significantly compared to the pristine graphene (κ = 225.59 W/mK), as shown in Figure S14b (ii). Note that introducing voids in graphene is one of the efficient methods to tune the graphene thermal conductivity; the void distribution followed the tubular-inspiration design can be a trade-off between the tunable thermal conductivity and decreased mechanical properties induced by voids. It is worth mentioning that the thermal conductivity of graphene is strain-dependent, which can be tuned by external mechanical deformation [12, 14]. Hence, to obtain a larger tunability range of graphene thermal conductivity, the stretchability (failure strain) of graphene should be improved to reach its full potential applications. While it is known that due to the brittle failure mechanism [15, 16], the stretchability of graphene and CNT is limited ($\varepsilon_f \approx 0.2$). The cellular-, gradient-, tubular-, layered-, and suture-inspired designs based on graphene illustrated in this work show increased failure strains compared to pristine graphene. Hence, these bioinspired designs can bring an extended tunability of thermal conductivity by external strains, which is worth further investigation.

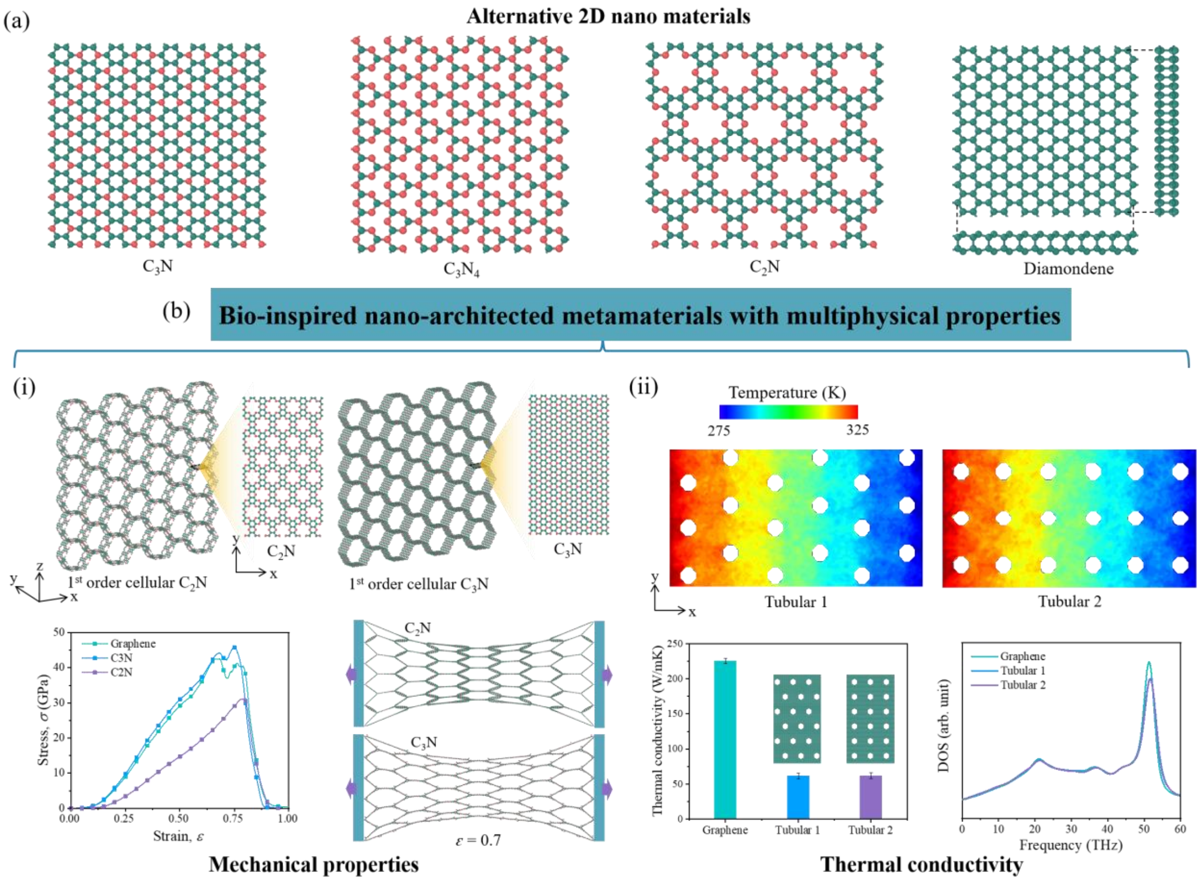

Figure S14. Base materials as building blocks and multiphysical properties of bioinspired nano-architected metamaterials. (a) Potential candidates of 2D nanomaterials for constructing bioinspired nano-architected metamaterials. (b) Multiphysical properties of bioinspired nano-architected metamaterials. (i) Cellular-inspired nano metamaterials constructed based on C_2N and C_3N and their corresponding stress-strain responses. The simulation temperature is set at 1 K all three samples. (ii) Thermal transport in tubular-inspired graphene metamaterials (The left and right sides are set at high and low temperature, respectively.) and their thermal conductivity and phonon density of states.

References

[1] Müller-Plathe, F., *The Journal of Chemical Physics* **1997**, 106 (14), 6082-6085. DOI 10.1063/1.473271.

[2] Zhang, W.; Yin, S.; Yu, T. X.; Xu, J., *Int J Impact Eng* **2019,** *125*, 163-172. DOI 10.1016/j.ijimpeng.2018.11.014.

[3] Mahmood, J.; Lee, E. K.; Jung, M.; Shin, D.; Jeon, I. Y.; Jung, S. M.; Choi, H. J.; Seo, J. M.; Bae, S. Y.; Sohn, S. D.; Park, N.; Oh, J. H.; Shin, H. J.; Baek, J. B., *Nat Commun* **2015,** *6*, 6486. DOI 10.1038/ncomms7486.

[4] Mahmood, J.; Lee, E. K.; Jung, M.; Shin, D.; Choi, H. J.; Seo, J. M.; Jung, S. M.; Kim, D.; Li, F.; Lah, M. S.; Park, N.; Shin, H. J.; Oh, J. H.; Baek, J. B., *Proc Natl Acad Sci U S A* **2016**, 113 (27), 7414-9. DOI 10.1073/pnas.1605318113.

[3] Mahmood, J.; Lee, E. K.; Jung, M.; Shin, D.; Jeon, I. Y.; Jung, S. M.; Choi, H. J.; Seo, J. M.; Bae, S. Y.; Sohn, S. D.; Park, N.; Oh, J. H.; Shin, H. J.; Baek, J. B., *Nat Commun* **2015,** *6*, 6486. DOI 10.1038/ncomms7486.

[4] Mahmood, J.; Lee, E. K.; Jung, M.; Shin, D.; Jeon, I. Y.; Jung, S. M.; Choi, H. J.; Seo, J. M.; Bae, S. Y.; Sohn, S. D.; Park, N.; Oh, J. H.; Shin, H. J.; Baek, J. B., *Nat Commun* **2015,** *6*, 6486. DOI 10.1038/ncomms7486.

[5] Algara-Siller, G.; Severin, N.; Chong, S. Y.; Bjorkman, T.; Palgrave, R. G.; Laybourn, A.; Antonietti, M.; Khimyak, Y. Z.; Krasheninnikov, A. V.; Rabe, J. P.; Kaiser, U.; Cooper, A. I.; Thomas, A.; Bojdys, M. J., *Angew Chem Int Ed Engl* **2014**, 53 (29), 7450-5. DOI 10.1002/anie.201402191.

[6] Gao, X.; Liu, H.; Wang, D.; Zhang, J., *Chem Soc Rev* **2019**, 48 (3), 908-936. DOI 10.1039/c8cs00773j.

[7] Cai, J.; Mi, C.; Deng, Q.; Zheng, C., *Mechanics of Materials* **2019**, 139. DOI 10.1016/j.mechmat.2019.103205.

[8] Ni, B.; Sinnott, S. B.; Mikulski, P. T.; Harrison, J. A., *Phys Rev Lett* **2002**, 88 (20), 205505. DOI 10.1103/PhysRevLett.88.205505.

[9] Fasolino, A.; Los, J. H.; Katsnelson, M. I., *Nat Mater* **2007**, 6 (11), 858-61. DOI 10.1038/nmat2011.

[10] Shen, L.; Shen, H.-S.; Zhang, C.-L., *Materials & Design* **2010**, 31 (9), 4445-4449. DOI 10.1016/j.matdes.2010.04.016.

[11] Yi, L.; Yin, Z.; Zhang, Y.; Chang, T., *Carbon* **2013**, 51, 373-380. DOI 10.1016/j.carbon.2012.08.069.

[12] Cai, J.; Estakhrianhaghighi, E.; Akbarzadeh, A., *Carbon* **2022**. DOI 10.1016/j.carbon.2022.02.008.

[13] Ghasemi, H.; Rajabpour, A.; Akbarzadeh, A. H., *International Journal of Heat and Mass Transfer* **2018**, 123, 261-271. DOI 10.1016/j.ijheatmasstransfer.2018.02.094.

[14] Cai, J.; Akbarzadeh, A., *Materials & Design* **2021**, 206. DOI 10.1016/j.matdes.2021.109811.

[15] Zhang, P.; Ma, L.; Fan, F.; Zeng, Z.; Peng, C.; Loya, P. E.; Liu, Z.; Gong, Y.; Zhang, J.; Zhang, X.; Ajayan, P. M.; Zhu, T.; Lou, J., *Nat Commun* **2014**, 5, 3782. DOI 10.1038/ncomms4782.

[16] Lee, C.; Wei, X.; Kysar, J. W.; Hone, J., *Science* **2008**, 321 (5887), 385-8. DOI 10.1126/science.1157996.

[17] Zhang, J., Nano Energy **2017**, 41, 460-468. DOI 10.1016/j.nanoen.2017.10.005.

[18] Guo, G. Y.; Ishibashi, S.; Tamura, T.; Terakura, K., *Physical Review B* **2007**, 75 (24). DOI 10.1103/PhysRevB.75.245403.

[19] Duerloo, K.-A. N.; Ong, M. T.; Reed, E. J., *The Journal of Physical Chemistry Letters* **2012**, 3 (19), 2871-2876. DOI 10.1021/jz3012436.

[20] Chen, X.; Wu, Z.; Xu, S.; Wang, L.; Huang, R.; Han, Y.; Ye, W.; Xiong, W.; Han, T.; Long, G.; Wang, Y.; He, Y.; Cai, Y.; Sheng, P.; Wang, N., *Nat Commun* **2015**, 6, 6088. DOI 10.1038/ncomms7088.

[21] Romano, G.; Mantini, G.; Di Carlo, A.; D'Amico, A.; Falconi, C.; Wang, Z. L., *Nanotechnology* **2011**, 22 (46), 465401. DOI 10.1088/0957-4484/22/46/465401.

[22] Zhang, J., *Applied Physics Letters* **2014**, 104 (25). DOI 10.1063/1.4885538.
